# Carnosic acid alleviates 5-Fluorouracil chemoresistance and enhances CD8^+^ T cell cytotoxicity in gastric cancer by regulating the CXCL12/CXCR7 axis

**DOI:** 10.3389/fimmu.2026.1925925

**Published:** 2026-09-08

**Authors:** Yinan Zhang, Dinuo Li, Xin Zhang

**Affiliations:** 1 Department of Neurology, The First Affiliated Hospital of Jinzhou Medical University, Jinzhou, China; 2 Department of General Surgery, The First Affiliated Hospital of Jinzhou Medical University, Jinzhou, China; 3 Department of Gastroenterology, The First Affiliated Hospital of Jinzhou Medical University, Jinzhou, China

**Keywords:** carnosic acid, CD8+ T cells, chemoresistance, CXCL12/CXCR7 axis, gastric cancer

## Abstract

**Background:**

Key features of gastric cancer (GC) include its aggressive nature, high metastatic capability, and a tendency for relapse. While 5-fluorouracil (5-FU) remains a cornerstone of GC treatment, its efficacy is substantially undermined by the emergence of acquired drug resistance. Carnosic acid (CA) has antitumor and immunomodulatory potential, but its role in 5-FU-resistant GC remains unclear. This study investigated whether CA enhances 5-FU sensitivity and CD8^+^ T cell-mediated antitumor immunity by regulating the CXCL12/CXCR7 axis.

**Methods:**

The effects of CA combined with 5-FU on cell viability, proliferation, apoptosis, migration, invasion, and EMT-related features were evaluated using 5-FU-sensitive and 5-FU-resistant GC cell models and MFC/MFC-R mouse GC models. CXCR7 overexpression, co-immunoprecipitation, immunofluorescence, and Western blotting were performed to examine CXCL12/CXCR7-axis regulation. GC cell/CD8^+^ T cell co-culture, subcutaneous tumor, lung metastasis, anti-PD-L1 combination therapy, and safety models were further used for validation.

**Results:**

5-FU-resistant GC cells showed reduced drug sensitivity and enhanced malignant phenotypes. CA plus 5-FU suppressed proliferation, migration, invasion, and EMT, while promoting apoptosis. Mechanistically, CA restored 5-FU sensitivity by inhibiting aberrant CXCL12/CXCR7-axis activation, whereas CXCR7 overexpression partially reversed these effects. The combined treatment also enhanced CD8^+^ T cell proliferation, cytotoxicity, and IFN-γ and Granzyme B expression, reduced immunosuppression-associated molecules, inhibited tumor growth and lung metastasis, potentiated anti-PD-L1 efficacy, and caused no obvious systemic toxicity.

**Conclusion:**

CA enhances 5-FU sensitivity, suppresses EMT and metastasis-associated phenotypes, and improves CD8^+^ T cell-mediated antitumor immunity in resistant GC by targeting the CXCL12/CXCR7 axis.

## Graphical Abstract

**Figure:**
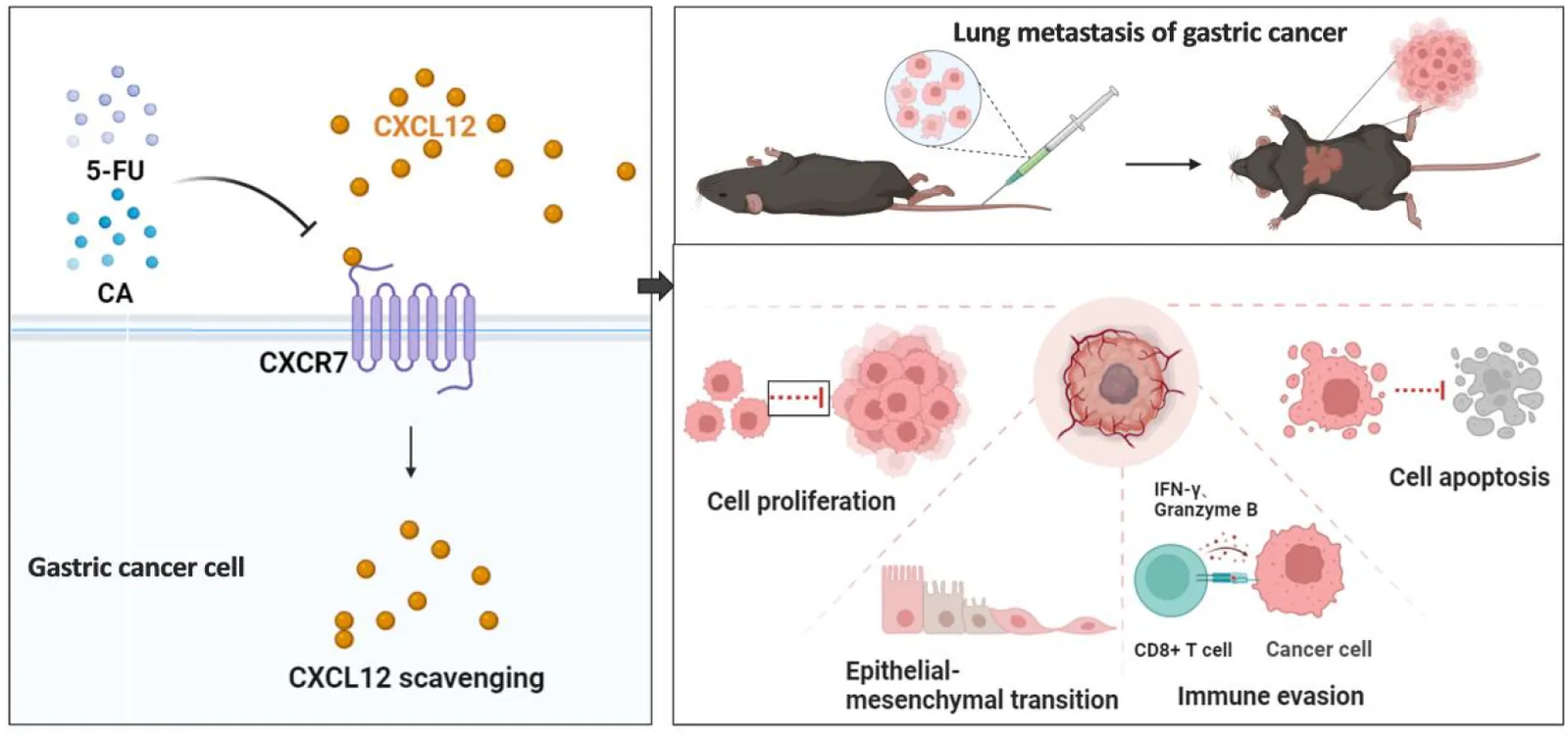
The susceptibility of drug-resistant gastric cancer cells to 5-FU exhibits potential for potentiation through the introduction of carnosic acid (CA). This effect may be associated with the inhibition of aberrant CXCL12/CXCR7 axis activation in resistant cells, thereby suppressing cell proliferation, epithelial–mesenchymal transition, and metastasis-associated phenotypes, while promoting apoptosis and enhancing CD8^+^ T cell-mediated antitumor immune responses.

## Highlights

- Gastric cancer cell susceptibility to 5-FU is markedly restored by carnosic acid (CA) through the elevation of chemosensitivity.
- The aggressive behaviors of drug-resistant gastric cancer cells, including proliferation, migration, invasion, and EMT, are concurrently suppressed via CA and 5-FU combination.
- CA effectively targets and alleviates 5-FU resistance-associated malignant phenotypes by inhibiting the CXCL12/CXCR7 signaling axis.
- The therapeutic potential of anti-PD-L1 treatment is synergistically augmented when coupled with CA and 5-FU, a benefit attributed to boosted CD8^+^ T cell cytotoxicity.

## 1 Introduction

Gastric cancer (GC) stands as a prominent digestive tract malignancy and a primary contributor to global cancer fatalities (1). GC often results in a sub-optimal prognosis, primarily because early-stage disease frequently remains asymptomatic. Despite ongoing evolution in diagnostic and therapeutic strategies, a significant proportion of patients is not identified until advanced stages of the disease (2). For patients with advanced GC or postoperative recurrence and metastasis, systemic chemotherapy remains an important therapeutic strategy (3). 5-fluorouracil (5-FU) stands as a cornerstone of the chemotherapeutic regimens routinely employed in the clinical management of GC (4). However, acquired resistance substantially limits the clinical efficacy of 5-FU in GC, with resistance eventually developing in most cases and reported median times to progression ranging from 0 to 8 months; notably, residual tumors after 5-FU-based neoadjuvant chemotherapy showed a higher BMI1-positive rate than untreated tumors (58.3% vs. 35.0%), which was further associated with poorer 3-year recurrence-free survival (HR = 2.455) (5). In addition to chemotherapy resistance, the limited response to immunotherapy remains another clinical challenge in advanced GC; in the phase III ORIENT-16 trial conducted in Chinese patients with advanced gastric or gastroesophageal junction adenocarcinoma, sintilimab plus chemotherapy achieved objective response rates of 58.2% in the overall population and 63.6% in patients with PD-L1 expression assessed by the combined positive score (CPS) ≥5 (6). Consequently, to optimize the clinical prognosis of GC, it is of paramount importance to unravel the molecular drivers of 5-FU refractoriness and explore innovative approaches for sensitizing tumor cells to chemotherapy.

The prime antitumor mechanisms of 5-FU revolve around the systematic disruption of essential cellular processes, specifically by inhibiting thymidylate synthase, impairing nucleic acid synthesis, and potentiating DNA damage (7). Nonetheless, enduring pharmacological stress compels GC cells to acquire resistance through various channels, such as diminished drug internalization, accelerated drug expulsion, fortified DNA repair, inhibited programmed cell death, and the stimulation of survival-centric signaling cascades (8). Consequently, endowing tumor cells with strengthened malignant survival advantages, this process is frequently characterized by the manifestation of other aggressive traits, including sustained proliferation, activated migration and invasion, and the onset of epithelial–mesenchymal transition (EMT) (9, 10). For GC cells, a strengthened tolerance toward chemotherapeutic agents may also result from the aberrant expression of resistance-associated molecules (11). Therefore, elucidating the mechanisms of 5-FU resistance from the perspectives of cell proliferation, death evasion, EMT activation, and regulation of drug resistance-related proteins is essential for improving the chemotherapeutic response of GC.

Apart from tumor cell-intrinsic resistance, the restructuring of the tumor’s immunological landscape has become a vital factor in governing the success of chemotherapy. Chemotherapy is capable of both eliminating tumor cells directly and regulating antitumor immune responses by regulating tumor antigen release, immune cell infiltration, and immune checkpoint expression (12). In the GC milieu, CD8^+^ T lymphocytes serve as the principal effector population, orchestrating the eradication of malignant cells through their potent cytolytic functions (13). Activated CD8^+^ T cells exert their lethal effects by discharging a variety of cytotoxic mediators. Key among these are lytic proteins, such as perforin and Granzyme B, alongside the potent anti-tumor cytokine interferon-γ (IFN-γ) (14). However, prolonged chemotherapeutic pressure and an immunosuppressive microenvironment can restrict CD8^+^ T cell proliferation, reduce cytotoxic activity, and induce an exhausted phenotype, thereby weakening their antitumor function (15). Furthermore, the overexpression of PD-L1 within the neoplastic compartment, alongside the amplification of negative regulatory signaling in T lymphocytes, collectively drives the process of immune subversion (16). Effectively counteracting chemoresistance in GC may rely on a dual-action approach: re-establishing CD8^+^ T cell-driven antitumor immunity in conjunction with amplifying the primary cytotoxic effects of 5-FU.

Chemokines and their cognate receptors are frequently implicated as pivotal contributors to multiple aggressive phenotypes, including tumor progression, metastatic dissemination, immune cell recruitment, and therapeutic resistance (17, 18). In this context, C-X-C motif chemokine ligand 12 (CXCL12), together with its receptor system, forms a central regulatory chemokine axis amidst the tumor microenvironment (19). By binding to receptors such as CXCR4 and CXCR7, CXCL12 regulates tumor cell migration, adhesion, proliferation, and remodeling of the immune microenvironment (20), and is also involved in chemoresistance in multiple malignancies (21). Among these receptors, CXCR7 has been closely associated with malignant progression in various tumors (22, 23). Importantly, clinical evidence from human GC tissues further supports the prognostic relevance of CXCR7. In a cohort of 150 patients with GC, high CXCR7 expression was significantly associated with lymph node metastasis and advanced TNM stage, while CXCR7-positive patients had a markedly shorter median overall survival than CXCR7-negative patients (40 vs. 75 months) (23). For tumor cells, a strengthened capacity to cope with therapeutic stress may also result from the pathological modulation of cell survival, invasive and metastatic dissemination, and drug response (24). Therefore, aberrant activation of the CXCL12/CXCR7 axis may contribute to malignant progression and therapeutic resistance by promoting 5-FU resistance and immune escape in GC.

In recent years, natural bioactive compounds have received growing attention for their potential roles in adjuvant cancer treatment and enhancement of chemosensitivity (25, 26). Because of their ability to act on multiple targets and their generally favorable toxicity profiles, natural products may help improve chemosensitivity and support better treatment tolerability (27). Carnosic acid (CA) is a natural phenolic diterpene compound with antioxidant, anti-inflammatory, and antitumor activities (28). It can modulate malignant tumor cell behaviors by regulating oxidative stress, cell cycle progression, apoptosis, and tumor-associated signaling pathways (29). Our previous study showed that CA inhibited the proliferation, migration, and invasion of gastric cancer AGS cells by regulating the CXCR7/CXCL12 axis (30). However, whether CA can reverse 5-FU resistance in GC by modulating the CXCL12/CXCR7 axis and enhance CD8^+^ T cell-mediated antitumor immunity remains to be systematically elucidated.

Based on the above background, we hypothesized that aberrant activation of the CXCL12/CXCR7 axis may promote 5-FU resistance, EMT, and immune escape in GC, whereas CA may enhance 5-FU sensitivity and restore CD8^+^ T cell-mediated antitumor immunity by inhibiting this axis. To test this hypothesis, we established 5-FU-resistant GC cell and animal models, and integrated functional assays, mechanistic validation, CD8^+^ T cell co-culture experiments, and anti-PD-L1 combination therapy models. This study systematically investigated the mechanism by which CA reverses 5-FU resistance and enhances antitumor immunity, providing experimental evidence for interventions targeting chemoresistance and for the optimization of chemo-immunotherapy strategies in GC.

## 2 Materials and methods

### 2.1 Reagents and chemicals

CA (purity ≥98%; MedChemExpress, USA; Cat# HY-N0644) was utilized to make a stock solution by dissolving it in dimethyl sulfoxide (DMSO; Sigma-Aldrich, USA; Cat# D2650). This resulting solution was then kept at −20 °C under light-protected conditions. Immediately preceding any *in vitro* experimentation, CA was further diluted into complete culture medium until the necessary final concentrations were achieved, ensuring that the DMSO concentration remained identical across all experimental groups. For the *in vivo* portion of the study, CA was formulated in phosphate-buffered saline (PBS) just prior to its intraperitoneal injection at the noted doses.

### 2.2 Cell culture and treatments

The human GC cell lines HGC27 (ECACC, UK; Cat# 94042256), MKN45 (JCRB Cell Bank, Japan; Cat# JCRB0254), BGC823 (Procell Life Science & Technology Co., Ltd., China; Cat# CL-0033), and AGS (ATCC, USA; Cat# CRL-1739), along with the human gastric mucosal epithelial cell line GES-1 (Cytion/CLS Cell Lines Service GmbH, Germany; Cat# 305428), were utilized throughout this investigation. Additionally, the study involved the murine GC cell line MFC (Procell Life Science & Technology Co., Ltd., China; Cat# CL-0156) and the murine gastric epithelial cell line GSM06 (RIKEN BioResource Research Center, Japan; Cat# RCB1779). To generate 5-FU-resistant variants, parental GC cells were subjected to prolonged stepwise exposure to increasing concentrations of 5-FU. The induction period for the human 5-FU-resistant GC cell lines was approximately 8 months, whereas MFC-R cells were generated over approximately 10 months. Following preliminary screening of 5-FU sensitivity, BGC823-R and MFC-R cells were selected as the primary resistant models for subsequent functional experiments and were maintained in medium containing 10 μg/mL and 0.5 μg/mL 5-FU, respectively. Before functional experiments, resistant cells were cultured in drug-free complete medium for 14 days to minimize the influence of acute 5-FU exposure. The stability of the resistant phenotype was verified by repeated CCK-8-based determination of IC_50_ values in parental and resistant cells. Maintenance of all cell lines was conducted at 37 °C in a humidified 5% CO_2_ atmosphere, using complete medium enriched with 10% fetal bovine serum (FBS; Gibco, USA; Cat# 10099-141) and 1% penicillin–streptomycin solution (Gibco, USA; Cat# 15140122). To maintain cell line integrity, we routinely verified each line using short tandem repeat (STR) profiling and conducted periodic screenings to confirm the absence of mycoplasma.

After a 24-hour incubation with a concentration range of 5-FU (0, 10, 20, 40, 80, 160, and 320 μg/mL), the viability of both parental and 5-FU-resistant GC lines was measured using the CCK-8 method, followed by the determination of IC50 values (31, 32). In a parallel effort to establish the appropriate intervention concentration for CA, the 5-FU-resistant GC cells and their normal gastric epithelial counterparts were treated with varying doses of CA (0, 5, 10, 20, 40, or 80 μg/mL) for 24 h, followed by CCK-8 analysis to monitor cell viability (33, 34). For the subsequent experiments, the working concentrations were established as follows: 40 μg/mL 5-FU for BGC823-R cells, 2.5 μg/mL 5-FU for MFC-R cells, and a uniform 20 μg/mL CA for both cell lines.

### 2.3 CXCR7 overexpression and experimental grouping

To explore how the CXCL12/CXCR7 signaling axis impacts 5-FU resistance and the potential chemosensitizing effects of CA in GC, a CXCR7 overexpression plasmid (OE-CXCR7) was generated alongside its empty vector control (OE-NC). Shanghai GenePharma Co., Ltd. handled the synthesis and design of both construct types. To achieve a confluence of approximately 60%–70% for transfection, BGC823-R and MFC-R cells were seeded into 6-well plates and incubated accordingly. At this point, the cells were transfected with the specified plasmids utilizing Lipofectamine 3000 (Invitrogen, USA; Cat# L3000015), adhering strictly to the provided manufacturer’s protocol. After 48 h of transfection, cells were collected to verify CXCR7 overexpression through immunoblotting, measuring the protein expression of both CXCR7 and its ligand CXCL12. For *in vivo* rescue experiments, MFC-R cells stably overexpressing CXCR7 and matched OE-NC control cells were established before implantation, and the overexpression efficiency was verified by Western blotting.

### 2.4 Cell viability assay

Cell viability was quantified using the Cell Counting Kit-8 (Beyotime, China; Cat# C0037). Briefly, cells were seeded at a concentration of 5 × 10³ per well into 96-well plates and permitted to adhere overnight. Subsequent to experimental interventions, each well was supplemented with 10 μL of CCK-8 reagent, followed by a 2-hour incubation at 37 °C protected from light. The resulting absorbance was measured at 450 nm with a Multiskan FC microplate reader (Thermo Fisher Scientific, USA). Cell viability was calculated according to the manufacturer’s instructions and expressed as cell viability (%).

### 2.5 Lactate dehydrogenase release assay

LDH leakage was assessed using a commercial LDH Cytotoxicity Assay Kit (Beyotime, China; Cat# C0017). After the experimental phase, culture media were collected and cleared of cellular debris by spinning at 300 × g for 5 min. The resulting supernatants were then mixed with the LDH detection cocktail as specified by the manufacturer’s protocol. Following a 30-minute reaction period at room temperature in the dark, absorbance was measured at 490 nm using a microplate reader. LDH release was calculated according to the manufacturer’s instructions and expressed as relative LDH release after normalization to the corresponding Control group.

### 2.6 Calcein AM/PI live-dead staining

Cell survival and cytotoxicity were assessed using a Calcein/PI Cell Viability/Cytotoxicity Assay Kit (Beyotime, China; Cat# C2015M). To perform live/dead cell visualization, cells were inoculated onto sterile coverslips within 24-well dishes. Following cell attachment and the completion of the assigned experimental treatments, the culture fluid was withdrawn, and samples were washed twice with PBS. Calcein AM/PI staining was then conducted for 30 minutes at 37 °C, ensuring light-protected conditions. After incubation, the coverslips were rinsed with PBS and visualized utilizing an Olympus IX73 fluorescence microscope (Olympus Corporation, Japan). For the assessment of live/dead cell percentages, five arbitrary fields per group were photographed and processed via ImageJ software.

### 2.7 EdU cell proliferation assay

Cell proliferative capacity was evaluated utilizing an EdU Kit (Beyotime, China; Cat# C0072S). Following treatment, cells were incubated with EdU for a 2-hour pulse, then fixed in 4% paraformaldehyde for 15 min. To allow for permeabilization, 0.3% Triton X-100 was applied for 10 min. The BeyoClick detection procedure was performed according to the provided manual, and nuclei were visualized by DAPI staining for 5 min. Fluorescence microscopy was utilized for imaging, and the ratio of EdU-positive cells was determined by evaluating five random fields for each group.

### 2.8 Colony formation assay

For clonogenic assessment, cells were deposited at a concentration of 500 per well within six-well culture plates. Once adherence was confirmed, the designated treatments were applied according to the experimental groups. The culture period spanned 14 days, during which the full growth medium was refreshed every 3 days. Upon the formation of visible colonies, the supernatants were discarded, followed by two PBS rinses. Fixation of the colonies (4% paraformaldehyde, 15 min) and subsequent 20-minute staining (0.1% crystal violet) were performed to prepare the samples for imaging. Only colonies containing more than 50 cells were counted.

### 2.9 Flow cytometric analysis of apoptosis

Apoptotic levels were assessed using the Annexin V-FITC/PI Detection Kit (Beyotime, China; Cat# C1062M). Following experimental interventions, cells were collected and underwent two sequential washes with PBS. The resulting cell pellets were resuspended in binding buffer to achieve a single-cell state before being labeled with Annexin V-FITC and PI. After a 15-minute incubation at room temperature in dark conditions, the apoptotic fractions were analyzed via flow cytometry (BD FACSCanto II, BD Biosciences, USA). Early and late apoptotic cells were distinguished and their percentages determined using FlowJo software; the summation of these two populations constituted the total apoptosis rate.

### 2.10 Wound healing assay

To evaluate cellular motility, a wound-healing protocol was implemented. Specifically, 6-well plates were seeded with 4 × 10^5^ cells per well and incubated until the monolayer reached approximately 90% density. A linear gap was introduced into the confluent cells using a sterile 200-μL pipette tip. After clearing dislodged cells with PBS, the culture environment was transitioned to serum-deprived medium. Microscopic observations were documented at the initial (0 h) and 24-h time points at identical coordinates. The migration efficiency was subsequently quantified by analyzing the wound area via ImageJ.

### 2.11 Transwell assay

Cell invasion was evaluated using 8-μm pore size Transwell inserts (Corning, USA; Cat# CLS3422). The upper chambers were pre-coated with 50 μL of Matrigel (Corning, USA; Cat# CLS354248) for invasion assays. Briefly, 5 × 10³ cells suspended in serum-free medium were seeded into the upper chamber, while medium containing 10% FBS was added to the lower chamber as a chemoattractant. After 24 h of incubation, cells remaining on the upper surface of the membrane were gently removed with a cotton swab. Cells that had invaded to the lower surface were fixed with 4% paraformaldehyde for 15 min and stained with 0.1% crystal violet for 20 min. After washing and air-drying, invaded cells were counted in five randomly selected microscopic fields.

### 2.12 Anoikis assay

To evaluate the capacity of GC cells for anchorage-independent survival upon extracellular matrix detachment, anoikis was assessed employing low-adhesion culture coupled with Annexin V-FITC/PI staining. Following the specified treatments, cells were introduced into ultra-low-attachment 6-well plates (Corning Costar, USA; Cat# 3471) for a 24 h suspension culture. Thereafter, the suspended cell populations were harvested, rinsed twice with PBS, and stained using an Annexin V-FITC Apoptosis Detection Kit (Beyotime, China; Cat# C1062M). The Annexin V-positive proportion was subsequently determined via flow cytometry. Analysis of early and late apoptotic cells was conducted utilizing FlowJo software, with the summation of these populations designated as the anoikis rate.

### 2.13 Immunofluorescence staining

For staining procedures, cells were seeded onto sterilized glass coverslips in 24-well plates at a concentration of 3 × 10^4^ cells/well and allowed to adhere overnight. Following experimental protocols, fixation was performed using 4% paraformaldehyde for 15 min, followed by a 10-minute membrane permeabilization with 0.1% Triton X-100. To minimize background interference, samples were subjected to a 1-hour blocking step with 5% BSA at room temperature. Overnight incubation at 4 °C followed, utilizing primary antibodies directed against E-cadherin (Cell Signaling Technology, USA; Cat# 3195; 1:1600), N-cadherin (Cell Signaling Technology, USA; Cat# 13116; 1:400–1:1600), CXCR7 (Sigma-Aldrich, USA; Cat# SAB4502446; 1:200), or CXCL12 (Proteintech, USA; Cat# 17402-1-AP; 1:200). The next day, cells were rinsed three times with PBS and incubated with Alexa Fluor 488-conjugated goat anti-rabbit IgG secondary antibody (Invitrogen, USA; Cat# A-11008; 1:500) for 1 h in the dark at room temperature. For visualization, nuclei were counterstained with DAPI (Beyotime, China; Cat# C1002). Five random fields per group were documented via fluorescence microscopy to visualize the stained cells. The resulting mean fluorescence intensity was analyzed via ImageJ software.

### 2.14 Western blot analysis

RIPA lysis buffer, fortified with protease and phosphatase inhibitors, was employed to isolate total protein from treated cells as well as murine tumor and lung specimens. Tissues were subjected to thorough homogenization prior to lysis, and all extraction procedures were conducted on ice. Post-centrifugation (12,000 × g, 15 min, 4 °C), the overlying liquid was recovered for the indicated assays. Quantification of protein concentrations was performed employing a BCA protein assay kit. For protein analysis, equivalent loads of protein were fractionated via SDS-PAGE and subsequently immobilized onto PVDF membranes that had been equilibrated in methanol. To eliminate background noise, a 1-hour blocking step was performed using 5% skimmed milk at room temperature. The membranes were then interrogated with specific primary antibodies overnight at 4 °C. After three successive 5-minute rinses in TBST, the blots were incubated with HRP-labeled secondary antibodies for 1 h. Protein signals were captured using an ECL substrate and quantified through densitometry with ImageJ software, employing GAPDH as the normalization reference.

Details regarding antibodies utilized for Western blotting throughout this study are presented in **Table 1**.

**Table 1:** Information on antibodies used in Western blot analysis.

Antibody	Source/catalog no.	Dilution
CXCR7	Abcam, UK, Cat# ab138509	1:1000
CXCL12/SDF-1	Human samples: Abcam, UK, Cat# ab9797 Mouse samples: Abcam, UK; Cat# ab25117	1:1000 1:1000
PD-L1	Abcam, UK, Cat# ab174838	1:1000
E-cadherin	Abcam, UK, Cat# ab231303	1:1000
EpCAM	Abcam, UK, Cat# ab32392	1:1000
N-cadherin	Abcam, UK, Cat# ab76011	1:1000
Vimentin	Abcam, UK, Cat# ab92547	1:1000
Snail	Abcam, UK, Cat# ab17732	1:1000
Twist1	Abcam, UK, Cat# ab50887	1:50
ZEB1	Abcam, UK, Cat# ab303480	1:1000
P-gp/ABCB1	Abcam, UK, Cat# ab168337	1:2000
MRP1/ABCC1	Abcam, UK, Cat# ab260038	1:1000
LRP	Abcam, UK, Cat# ab273093	1:1000
IFN-γ	Human samples: Abcam, UK, Cat# ab9657 Mouse samples: Abcam, UK; Cat# ab216642	1:1000 1:500
Granzyme B	Abcam, UK, Cat# ab255598	1:1000
PD-1	Human samples: Abcam, UK, Cat# ab237727 Mouse samples: Abcam, UK; Cat# ab309360	1:1000
CTLA4	Abcam, UK, Cat# ab134090	1:5000
LAG-3	Human samples: Abcam, UK, Cat# ab307517 Mouse samples: Abcam, UK; Cat# ab209238	1:1000
GAPDH	Abcam, UK, Cat# ab181602	1:10000
Goat anti-Rabbit IgG-HRP	Abcam, UK, Cat# ab97051	1:5000
Goat anti-Mouse IgG-HRP	Abcam, UK, Cat# ab97040	1:2000

### 2.15 Co-immunoprecipitation assay

To investigate protein interactions, cells subject to the indicated treatments were lysed on ice utilizing pre-chilled non-denaturing Cell Lysis Buffer (Abcam, UK; Cat# ab152163). Supernatants were collected following centrifugation (12,000 × g, 15 min, 4 °C). While a portion of the total protein lysate was set aside as the Input control, the remainder was subjected to immunoprecipitation. This involved overnight incubation at 4 °C with Protein A/G magnetic beads (Abcam, UK; Cat# ab286842) and the corresponding antibodies: anti-CXCR7 (Abcam, UK; Cat# ab138509), anti-CXCL12/SDF-1 (human samples: Abcam, UK; Cat# ab9797; mouse samples: Abcam, UK; Cat# ab25117), or rabbit IgG isotype control (Abcam, UK; Cat# ab172730). The following day, the magnetic beads underwent washing, were resuspended in protein loading buffer, and denatured by boiling. The resulting precipitates were subsequently analyzed via immunoblotting to identify the existence of CXCR7 and CXCL12.

### 2.16 Preparation and activation of human and mouse CD8^+^ T cells

Human primary CD8^+^ T cells were purchased from STEMCELL Technologies (Canada; Cat# 70027). Splenic lymphocytes from 615 mice served as the source for mouse CD8^+^ T cells. Under aseptic conditions, spleens were harvested and placed in pre-cooled RPMI 1640 medium, mechanically dissociated, and passed through a 70-μm cell strainer to obtain single-cell suspensions. After centrifugation, the supernatant was removed, and the cells were treated with red blood cell lysis buffer (Beyotime, China; Cat# C3702) for 2 min at room temperature. Complete medium was then added to terminate erythrocyte lysis, followed by washing with PBS. Mouse CD8^+^ T cells were enriched by negative magnetic selection using a Mouse CD8a^+^ T Cell Isolation Kit (Miltenyi Biotec, Germany; Cat# 130-104-075). The purity of the isolated CD8^+^ T cells was assessed by flow cytometry and reached 92.5%.

Following preparation, both human and murine CD8^+^ T cells were maintained in complete RPMI-1640 medium. Activation was accomplished via stimulation with anti-CD3 and anti-CD28 antibodies for 48 h. Specific stimulation parameters were as follows: for human CD8^+^ T cells, anti-human CD3 (Abcam, UK; Cat# ab210317; 2 μg/mL) and anti-human CD28 (Cell Signaling Technology, USA; Cat# 91920; 1 μg/mL) antibodies. Mouse CD8^+^ T cells were cultured in RPMI 1640 medium supplemented with 10% FBS, 1% penicillin–streptomycin, and 100 U/mL recombinant mouse IL-2, and were activated for 72 h using plate-bound anti-mouse CD3 antibody (Abcam, UK; Cat# ab174589; 1 μg/mL) together with soluble anti-mouse CD28 antibody (Cell Signaling Technology, USA; Cat# 34596; 2 μg/mL). Post-activation, CD8^+^ T cells were utilized in subsequent co-culture assays with GC cells.

### 2.17 Co-culture of GC cells and CD8^+^ T cells

To explore cellular interactions, a co-culture assay involving GC cells and CD8^+^ T cells was established. Following specified treatments according to the experimental grouping, the GC cells were incubated with activated CD8^+^ T cells of the corresponding species. This co-culture was maintained at a cell ratio of 1:10 (tumor cells to CD8^+^ T cells) for a duration of 48 h. Subsequently, GC cells and CD8^+^ T cells were harvested individually for further downstream analyses.

### 2.18 CFSE assay for CD8^+^ T cell proliferation

To monitor CD8^+^ T cell expansion, we employed a CFDA-SE Cell Proliferation and Tracking Kit (Beyotime, China; Cat# C0051). Isolated and activated CD8^+^ T cells were washed in PBS and resuspended at a concentration of 1 × 10^6^ cells/mL. Labeling was performed by applying CFDA-SE working solution (5 μM final concentration) for 15 min at 37 °C while shielded from light. The staining process was then terminated using complete medium. After co-culturing the labeled lymphocytes with treated GC cells, the non-adherent immune cells were collected for flow cytometric measurement of CFSE dilution. Data analysis was subsequently performed with FlowJo software.

### 2.19 Subcutaneous tumor model and drug administration

For the *in vivo* investigations, male 615 mice (aged 4–6 weeks) were acquired from Cavens Biotechnology Co., Ltd. (Nanjing, China). Specific pathogen-free (SPF) housing was provided for all animals under the following standardized conditions: 22 ± 2 °C temperature, 50%–60% relative humidity, and a 12-h light/dark cycle. Mice had ad libitum access to water and sustenance. An acclimatization period of at least 1 week was allowed before any experimental procedures. Compliance with all ethical standards was ensured, and the study was approved by the Experimental Animal Ethics Committee.

To create a subcutaneous model, MFC-R cells were introduced into the mice’s axillary region via subcutaneous injection at a dose of 5 × 10^5^ cells in 100 μL per mouse. Following tumor formation, mice were allocated into varied treatment groups (six mice per group) through a randomization process. Drug interventions consisted of: intraperitoneal injection of 5-FU (20 mg/kg, every 2 days), and intraperitoneal injection of CA (20 mg/kg, every 2 days). In the specific anti-PD-L1 combination therapy experiment, mice received anti-PD-L1 antibody (200 μg/mouse, twice weekly) added to the 5-FU and/or CA regimen. The corresponding control populations were administered an equivalent dose of isotype IgG or an equal volume of vehicle. For *in vivo* CXCR7 rescue experiments, mice were inoculated with MFC-R/OE-NC or MFC-R/OE-CXCR7 cells, followed by 5-FU+CA treatment according to the same dosing schedule described above. The progression of tumor size was periodically assessed using calipers to record the major and minor axes, and volumes were estimated according to the equation: V = 0.5 × long diameter × short diameter². At the predefined experimental conclusion, tumors were harvested and their mass was determined following the euthanasia of the mice.

### 2.20 Lung metastasis model

To examine the impact of CA on GC metastasis, a mouse lung metastasis model was established. Mice were assigned to the indicated groups, and the corresponding cell suspensions were injected intravenously via the tail vein at a density of 3 × 10^5^ cells in 100 μL PBS per mouse. Following injection, mice received the indicated drug treatments according to the experimental protocol. At the end of the four-week period, mice were humanely euthanized, and lung tissues were collected for subsequent analysis.

### 2.21 Hematoxylin and eosin staining

For histological analysis, mouse tissues (tumor, lung, liver, and kidney) were subjected to fixation with 4% paraformaldehyde (overnight). The specimens were subjected to dehydration through an escalating ethanol gradient, followed by clearing with xylene and infiltration with paraffin. Subsequently, 4-μm-thick slides were prepared by sectioning the resulting paraffin blocks. Post-sectioning, the slides underwent deparaffinization via two xylene treatments (10 min each) and rehydration through decreasing ethanol concentrations (5 min per step), followed by a rinse in distilled water. The standard H&E staining protocol was then initiated. Initial staining with hematoxylin for nuclei was followed by a running water rinse, differentiation, and bluing. For counterstaining, eosin was utilized. In the final step, the stained sections were subjected to dehydration via an ascending ethanol gradient and cleared with xylene, followed by mounting in neutral resin. Histopathological morphology was visualized and captured utilizing a light microscope.

### 2.22 TUNEL assay

To determine the proportion of apoptotic cells, the TUNEL Apoptosis Detection Kit (Invitrogen, USA; Cat# C10625) was employed. Paraffin-embedded sections initially underwent deparaffinization via two xylene treatments (10 min each), followed by hydration using decreased ethanol concentrations (5 min per concentration). To facilitate tissue permeability, proteinase K treatment was conducted for 15 min. Subsequent TUNEL staining proceeded at 37 °C for 1 h in a light-protected environment. Subsequent to the staining procedure, DAPI was applied for nuclear counterstaining. Fluorescence microscopy was then utilized to observe localization, during which five random fields were recorded for quantitative assessment.

### 2.23 Immunohistochemistry

To examine protein expression in paraffin-embedded tissues, sections were initially dewaxed and rehydrated. Heat-induced antigen unmasking was achieved in a sodium citrate solution, with slides gradually returning to room temperature via passive cooling. To neutralize internal peroxidase enzymes, sections were soaked in 3% H_2_O_2_ for 10 min. Non-specific interactions were suppressed by a 30-min blocking phase with 5% BSA. The specimens were then refrigerated overnight at 4 °C with the primary antibody. Following PBS washes, HRP-labeled secondary antibodies were applied for 60 min at room temperature before DAB-mediated color development. After counterstaining with hematoxylin, the slides underwent dehydration through graded ethanol, clarification in xylene, and final mounting. Brightfield microscopy was utilized for image acquisition. From each group, five random high-power fields were evaluated to quantify the positive staining area or percentage of positive cells utilizing ImageJ software.

Primary and secondary antibodies used for IHC are listed in **Table 2**.

**Table 2:** Information on antibodies used for IHC.

Antibody	Source/catalog no.	Dilution
Ki67	Abcam, UK, Cat# ab16667	1:200
CD8	Abcam, UK; Cat# ab209775	1:2000
E-cadherin	Abcam, UK, Cat# ab231303	1 μg/mL
N-cadherin	Abcam, UK, Cat# ab76011	1:50
Goat anti-Mouse IgG-HRP	Abcam, UK, Cat# ab205719	1:10000
Goat anti-Rabbit IgG-HRP	Abcam, UK, Cat# ab97051	1:500

### 2.24 Flow cytometric analysis of tumor-infiltrating CD8^+^ cells

To quantify the frequency of tumor-infiltrating CD8^+^ cells, fresh neoplastic tissues were mechanically minced and subjected to enzymatic dissociation at 37 °C to generate single-cell suspensions. The resulting mixtures were passed through 70-μm cell strainers and rinsed with PBS. After eliminating erythrocytes using a lysis buffer, the cell pellets were washed and adjusted to the required concentration. Following Fc receptor blocking, cells were stained with an APC-conjugated anti-mouse CD8a antibody (Abcam, UK; Cat# ab25499; 0.25 μg/test) according to the manufacturer’s instructions. Data were acquired by flow cytometry and analyzed using FlowJo software, with results expressed as the percentage of CD8^+^ cells in tumor tissues.

### 2.25 Safety evaluation of CA in mice

To explore the *in vivo* safety profile of CA, mice were allocated into Control and CA experimental cohorts. Body weight was recorded every 7 days during the 4-week treatment period. Upon reaching the designated study endpoint, blood samples were harvested and incubated at room temperature, with subsequent centrifugation performed to isolate the serum. Serum quantification of AST, ALT, BUN, and creatinine (CRE) levels followed, employing specified assay kits according to the manufacturers’ directives: AST (Elabscience, USA; Cat# E-BC-K008-M), ALT (Elabscience, USA; Cat# E-BC-K007-M), BUN (Elabscience, USA; Cat# E-BC-K013-M), and CRE (Elabscience, USA; Cat# E-BC-K010-S).

### 2.26 Statistical analysis

Quantitative data in this study are expressed as the mean ± standard deviation (mean ± SD). *In vitro* assays were performed with at least n ≥ 3 independent biological replicates, while *in vivo* cohorts consisted of n = 6 animals each. Statistical quantification and visualization were achieved using GraphPad Prism 9.0. To evaluate differences, a two-tailed unpaired Student’s t-test was employed for pairwise comparisons. For studies involving multiple groups, one-way analysis of variance (one-way ANOVA) followed by a Tukey’s *post hoc* test was applied. Statistical significance was defined at a threshold of *P* < 0.05.

## 3 Results

### 3.1 CA enhances the cytotoxic effect of 5-FU and inhibits proliferation in 5-FU-resistant GC cells

**Figure 1:**
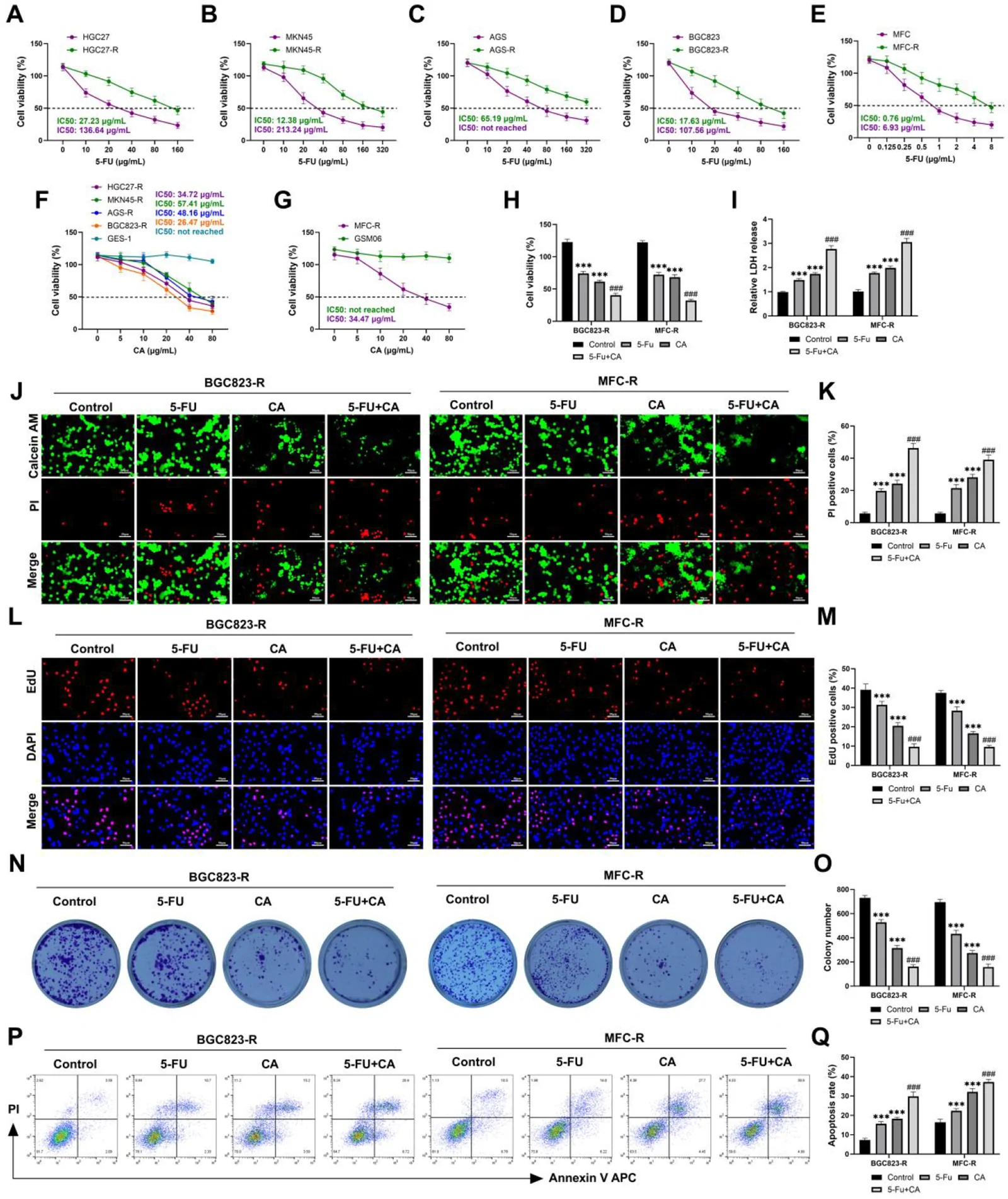
CA enhances the cytotoxic effect of 5-FU on 5-FU-resistant GC cells and suppresses their proliferation. **(A–D)** CCK-8 assays showed that human 5-FU-resistant GC cells exhibited reduced sensitivity to 5-FU compared with their parental cells. **(E)** MFC-R cells showed stronger tolerance to 5-FU than MFC cells. **(F, G)** CA exerted relatively weak effects on normal gastric epithelial cells, including GES-1 and GSM06 cells, while inhibiting the viability of 5-FU-resistant GC cells. **(H, I)** CA combined with 5-FU further reduced the viability of BGC823-R and MFC-R cells and increased LDH release. **(J, K)** Calcein AM/PI staining showed that the combined treatment increased the proportion of dead cells. **(L–O)** EdU and colony formation assays revealed that the joint treatment resulted in a more pronounced suppression of cell proliferation and clonogenic potential. **(P, Q)** Flow cytometric analysis demonstrated that the combination of CA and 5-FU triggered increased apoptosis in 5-FU-resistant GC cells. Data are presented as the mean ± SD. ^***^P < 0.001 vs. Control; ^###^*P* < 0.001 vs. 5-FU.

The sensitivity of 5-FU-resistant GC cells to 5-FU was assessed using CCK-8 assays by comparing the dose-dependent effects of 5-FU on parental cell lines, including HGC27, MKN45, AGS, and BGC823 cells, and their corresponding 5-FU-resistant counterparts. Cell viability decreased progressively with increasing 5-FU concentrations in all groups. Compared with parental cells, the resistant cells maintained higher viability and exhibited increased IC50 values, indicating the successful establishment of 5-FU-resistant cell models (**Figures 1A–D**). Similarly, MFC-R cells showed lower sensitivity to 5-FU than parental MFC cells (**Figure 1E**).

The dose-dependent effects of CA on 5-FU-resistant GC cells were then examined in parallel with normal gastric epithelial cells. CA exerted relatively limited effects on the viability of normal gastric epithelial cells, including GES-1 and GSM06 cells, whereas it reduced the viability of human 5-FU-resistant GC cells and MFC-R cells in a concentration-dependent manner (**Figures 1F, G**).

Further functional assays showed that the viability of BGC823-R and MFC-R cells was reduced by either 5-FU or CA treatment. Compared with either monotherapy, the combination of 5-FU and CA exerted a stronger inhibitory effect on cell viability and triggered robust LDH leakage (**Figures 1H, I**). These results were further supported by Calcein AM/PI staining, which showed a marked reduction in viable cells and an increase in dead cells after combined treatment (**Figures 1J, K**). EdU staining and colony formation assays further demonstrated that CA combined with 5-FU more strongly suppressed the proliferative and colony-forming abilities of resistant cells (**Figures 1L–O**). In addition, flow cytometric analysis showed that the combination treatment markedly increased the apoptosis rate of BGC823-R and MFC-R cells (**Figures 1P, Q**).

Taken together, these findings suggest that CA potentiates the cytotoxic effect of 5-FU in chemoresistant GC cells, accompanied by reduced cell proliferation, impaired clonogenic capacity, and increased apoptosis.

### 3.2 CA enhances 5-FU-mediated suppression of migration, invasion, and EMT in 5-FU-resistant GC cells

**Figure 2:**
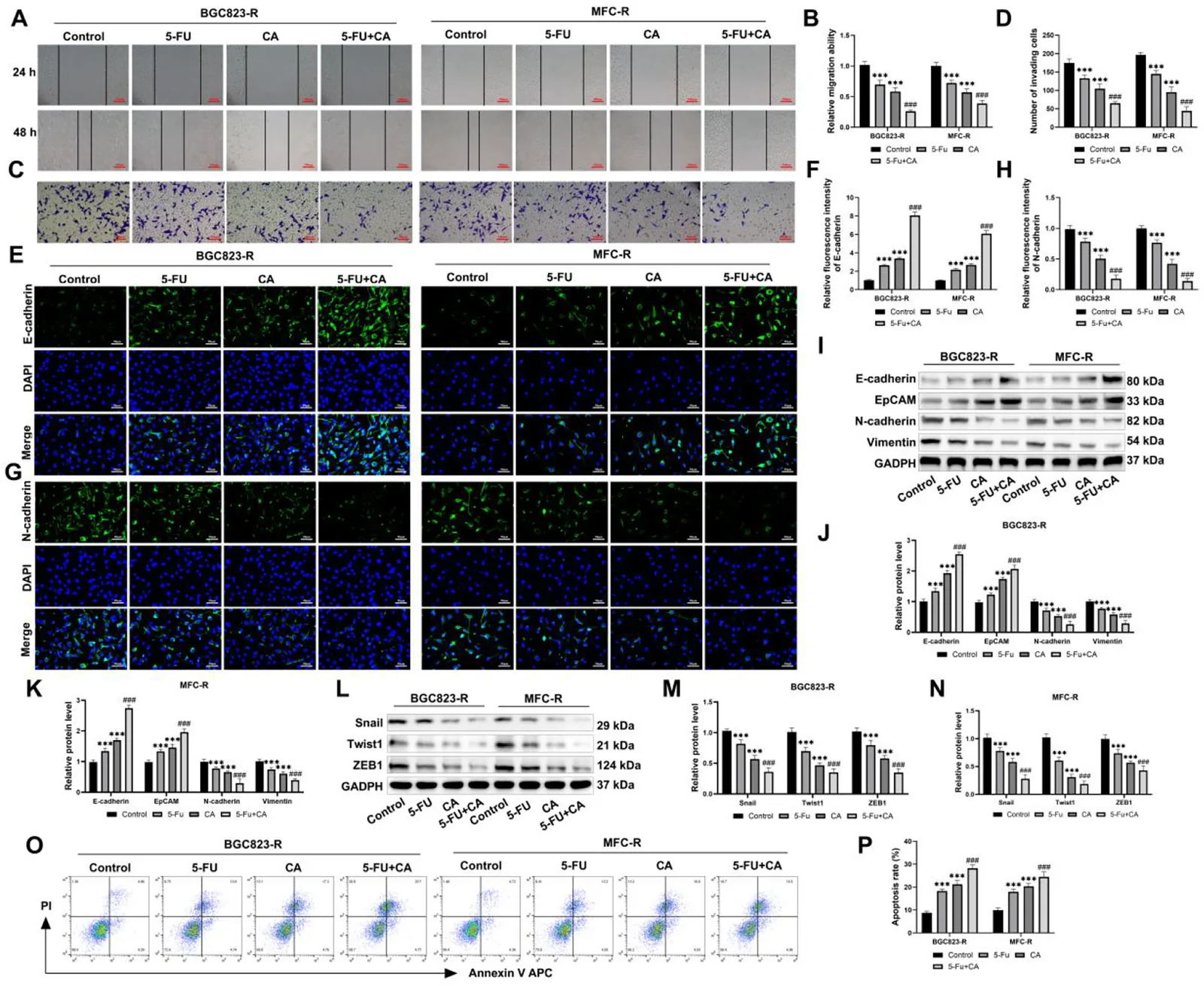
CA combined with 5-FU suppresses migration, invasion, and EMT and promotes anoikis in 5-FU-resistant GC cells. **(A, B)** Wound healing assays showed that CA combined with 5-FU further reduced the migratory ability of BGC823-R and MFC-R cells. **(C, D)** Transwell assays demonstrated that the combined regimen decreased the quantity of invading resistant cells. **(E–H)** Immunofluorescence imaging demonstrated that CA in conjunction with 5-FU enhanced E-cadherin levels while reducing N-cadherin expression. **(I–K)** Western blot analysis revealed that the joint treatment upregulated the expression of E-cadherin and EpCAM, but diminished the levels of N-cadherin and Vimentin. **(L–N)** CA combined with 5-FU further downregulated the expression of Snail, Twist1, and ZEB1. **(O, P)** Flow cytometric analysis revealed that the joint treatment elevated the percentage of anoikis in resistant cells. Data are presented as the mean ± SD. ^***^P < 0.001 *vs.* Control; ^###^*P* < 0.001 *vs.* 5-FU.

To determine whether CA enhanced the inhibitory effect of 5-FU on migration and invasion, BGC823-R and MFC-R cells were subjected to wound healing and Transwell assays. The Control group showed obvious wound closure, whereas 5-FU treatment reduced wound healing ability. This inhibitory effect on cell migration was further strengthened by CA combined with 5-FU (**Figures 2A, B**). Consistently, Transwell assays showed that 5-FU reduced the number of invading cells, and this effect was further enhanced by CA treatment (**Figures 2C, D**).

EMT-related markers were then examined by immunofluorescence staining and Western blotting. Immunofluorescence staining showed that Control cells had relatively low E-cadherin expression and high N-cadherin expression. 5-FU treatment increased E-cadherin expression and decreased N-cadherin expression, and these changes were more evident in the 5-FU+CA group (**Figures 2E–H**). Western blot analysis further showed that 5-FU increased the protein levels of E-cadherin and EpCAM, while reducing the levels of N-cadherin and Vimentin. These effects were further enhanced by combined treatment with CA and 5-FU (**Figures 2I–K**). In addition, the expression levels of the EMT-related transcription factors Snail, Twist1, and ZEB1 were further decreased after combined treatment, suggesting that CA enhanced the inhibitory effect of 5-FU on the EMT program (**Figures 2L–N**).

Further anoikis analysis showed that resistant cells in the Control group retained strong survival capacity under suspension culture. 5-FU increased the proportion of anoikis, and this effect was further enhanced by CA treatment (**Figures 2O, P**). Together, these results indicate that CA enhances the 5-FU-mediated inhibition of migration, invasion, and EMT in chemoresistant GC cells, accompanied by increased anoikis.

### 3.3 CA combined with 5-FU suppresses 5-FU-resistant GC cells and improves CD8^+^ T cell function in the co-culture system

**Figure 3:**
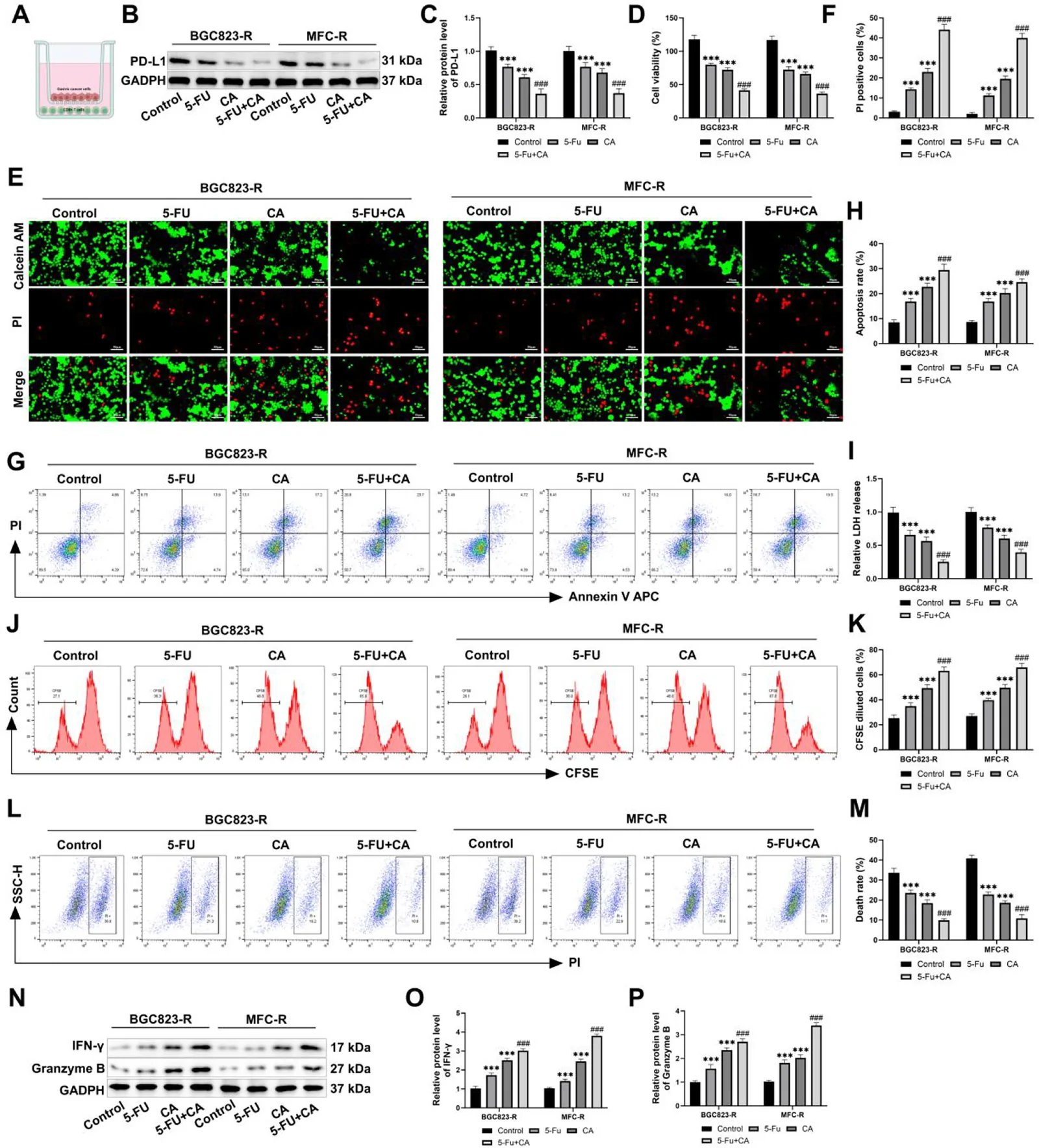
CA combined with 5-FU suppresses tumor cell survival and improves CD8^+^ T cell function in the co-culture system. **(A)** Schematic illustration of the co-culture system involving 5-FU-resistant GC cells and CD8^+^ T cells. **(B, C)** Western blot analysis showed that CA combined with 5-FU downregulated PD-L1 protein expression in tumor cells. **(D)** CCK-8 assays showed that CA combined with 5-FU further reduced the viability of tumor cells in the co-culture system. **(E, F)** Calcein AM/PI staining showed that the combined treatment increased tumor cell death. **(G, H)** Flow cytometric analysis showed that CA combined with 5-FU further increased tumor cell apoptosis. **(I)** LDH release assays showed that CA combined with 5-FU reduced LDH release from CD8^+^ T cells, suggesting decreased CD8^+^ T cell injury. **(J, K)** CFSE assays showed that CA combined with 5-FU promoted CD8^+^ T cell proliferation. **(L, M)** Flow cytometric analysis showed that CA combined with 5-FU reduced CD8^+^ T cell apoptosis. **(N–P)** Western blot analysis showed that CA combined with 5-FU upregulated IFN-γ and Granzyme B protein expression in CD8^+^ T cells, indicating enhanced CD8^+^ T cell effector function. Data are presented as the mean ± SD. ^***^P < 0.001 *vs.* Control; ^###^*P* < 0.001 *vs.* 5-FU.

To assess whether CA combined with 5-FU enhances antitumor immune activity, a co-culture model consisting of 5-FU-resistant GC cells and CD8^+^ T lymphocytes was established (**Figure 3A**). Western blot analysis showed that resistant GC cells maintained relatively high PD-L1 expression under co-culture conditions, whereas CA combined with 5-FU further reduced PD-L1 protein levels in tumor cells, suggesting that CA may attenuate the immunosuppressive phenotype of resistant cells (**Figures 3B, C**).

Within the co-culture system, CCK-8 analysis showed that 5-FU alone reduced the viability of resistant GC cells, and the addition of CA further strengthened this inhibitory effect (**Figure 3D**). Consistently, Calcein AM/PI staining showed a marked decrease in viable tumor cells and an increase in dead tumor cells in the 5-FU+CA group (**Figures 3E, F**). Flow cytometric analysis further showed that tumor cell apoptosis was markedly increased after combined treatment compared with 5-FU monotherapy, indicating that CA enhanced the antitumor effect of 5-FU in the co-culture system (**Figures 3G, H**).

The functional status of CD8^+^ T cells was then evaluated. LDH assays showed that CA combined with 5-FU reduced LDH release from CD8^+^ T cells, suggesting decreased CD8^+^ T cell injury under co-culture conditions (**Figure 3I**). CFSE assays showed that CD8^+^ T cell proliferation was increased in the 5-FU+CA group, as reflected by enhanced CFSE fluorescence dilution (**Figures 3J, K**). Meanwhile, flow cytometric analysis showed that the combined treatment reduced the apoptosis rate of CD8^+^ T cells, indicating improved CD8^+^ T cell survival (**Figures 3L, M**). Western blot analysis further demonstrated that CA combined with 5-FU upregulated IFN-γ and Granzyme B protein expression in CD8^+^ T cells, suggesting enhanced effector function (**Figures 3N–P**).

In summary, these findings indicate that CA combined with 5-FU not only promotes the death of chemoresistant GC cells in the co-culture system but also supports CD8^+^ T cell proliferation, survival, and effector function, thereby improving antitumor immune activity.

### 3.4 CA combined with 5-FU suppresses CXCL12/CXCR7 axis activation in 5-FU-resistant GC cells

**Figure 4:**
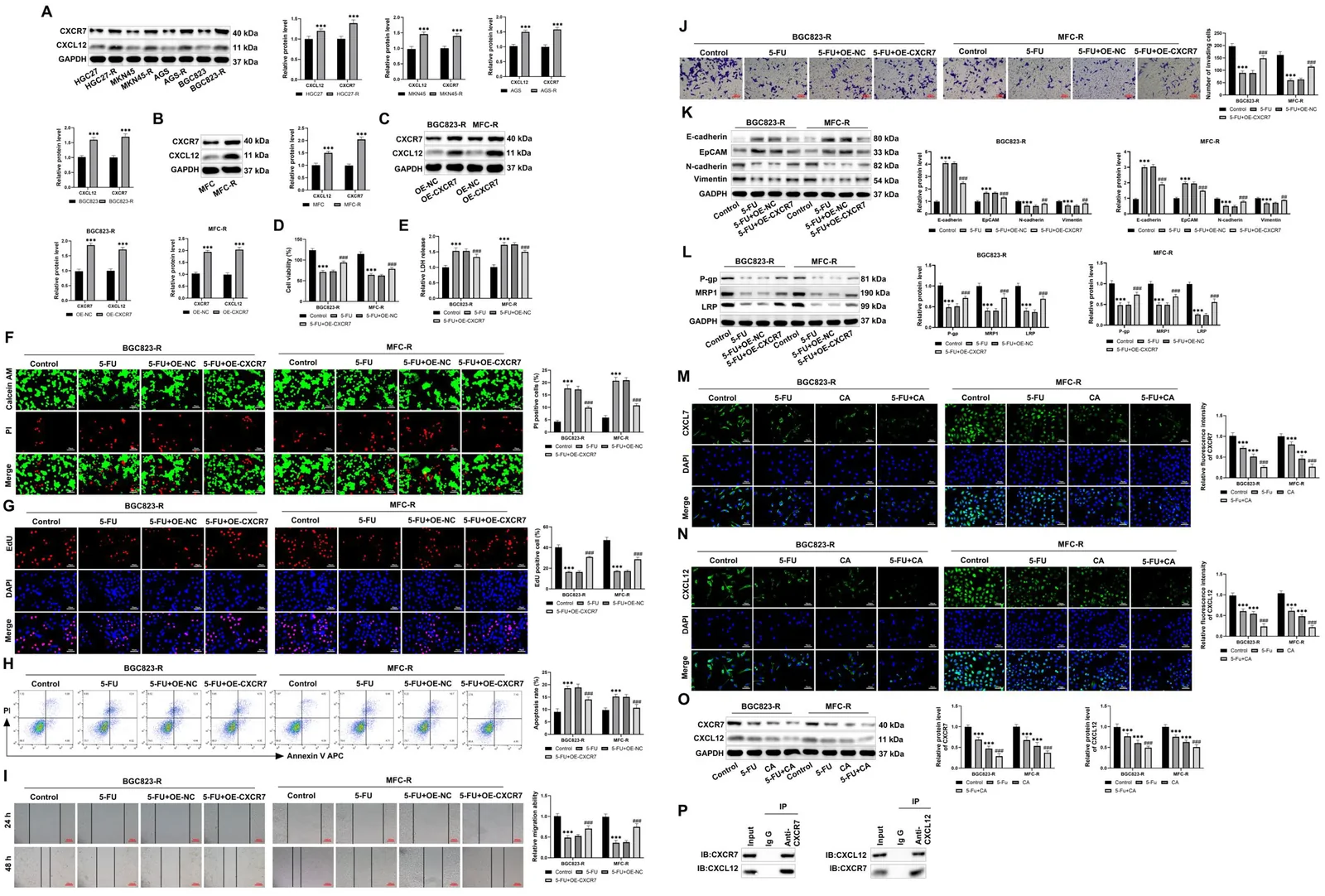
Activation of the CXCL12/CXCR7 axis promotes 5-FU resistance in GC, whereas CA combined with 5-FU suppresses this axis. **(A, B)** Western blot analysis showed that CXCR7 and CXCL12 expression levels were higher in 5-FU-resistant GC cells than in parental cells. **(C)** CXCR7 overexpression upregulated the protein expression of CXCR7 and CXCL12. **(D–H)** CXCR7 overexpression partially restored cell viability and proliferative capacity after 5-FU treatment and reduced cell injury, cell death, and apoptosis. **(I, J)** CXCR7 overexpression enhanced the migration and invasion of resistant cells after 5-FU treatment. **(K, L)** CXCR7 overexpression promoted maintenance of the EMT phenotype and upregulated drug resistance-related proteins, including P-gp, MRP1, and LRP. **(M–O)** Immunofluorescence staining and Western blot analysis showed that CA combined with 5-FU reduced CXCR7 and CXCL12 expression. **(P)** Co-immunoprecipitation confirmed an interaction between CXCR7 and CXCL12. Data are presented as the mean ± SD. ^***^P < 0.001 *vs.* Control; ^###^*P* < 0.001 *vs.* 5-FU+OE-NC or 5-FU.

The potential contribution of the CXCL12/CXCR7 axis to 5-FU resistance in GC was subsequently examined. Western blot analysis showed that, compared with the parental HGC27, MKN45, AGS, and BGC823 cells, their corresponding resistant cells, including HGC27-R, MKN45-R, AGS-R, and BGC823-R cells, exhibited markedly increased protein levels of CXCR7 and CXCL12 (**Figure 4A**). In the mouse cell system, CXCR7 and CXCL12 expression levels were also higher in MFC-R cells than in MFC cells (**Figure 4B**), suggesting that this axis may be associated with 5-FU resistance in GC.

To evaluate the functional role of CXCR7, BGC823-R and MFC-R cells were engineered to overexpress this receptor. Western blot analysis showed that CXCR7 expression was significantly increased in the OE-CXCR7 group, accompanied by elevated CXCL12 levels, suggesting upregulation of the CXCL12/CXCR7 axis (**Figure 4C**). Under 5-FU treatment, CXCR7 overexpression partially restored cell viability, reduced LDH release, cell death, and apoptosis, and increased the proportion of EdU-positive cells, suggesting that CXCR7 activation attenuated the cytotoxic effect of 5-FU on resistant cells (**Figures 4D–H**).

Further functional experiments showed that CXCR7 overexpression enhanced the migratory and invasive abilities of 5-FU-treated resistant GC cells (**Figures 4I, J**). Western blot analysis revealed that forced CXCR7 expression decreased E-cadherin and EpCAM levels while increasing N-cadherin and Vimentin expression, suggesting that CXCR7 helped maintain the EMT phenotype under 5-FU treatment (**Figure 4K**). Furthermore, CXCR7 overexpression elevated the protein levels of the chemoresistance-associated markers P-gp, MRP1, and LRP, consistent with enhanced drug-resistant characteristics (**Figure 4L**).

The effect of CA combined with 5-FU on the CXCL12/CXCR7 axis was then investigated. Immunofluorescence staining showed strong CXCR7 and CXCL12 signals in resistant cells in the Control group, whereas both fluorescence signals were markedly weakened in the 5-FU+CA group (**Figures 4M, N**). Western blot analysis further confirmed that CA combined with 5-FU significantly downregulated CXCR7 and CXCL12 protein levels (**Figure 4O**). Co-immunoprecipitation results confirmed an interaction between CXCL12 and CXCR7 (**Figure 4P**).

Collectively, these results demonstrate that activation of the CXCL12/CXCR7 axis contributes to 5-FU resistance in GC, whereas CA combined with 5-FU suppresses this axis in 5-FU-resistant GC cells.

### 3.5 CA restores 5-FU sensitivity in resistant GC cells by suppressing the CXCL12/CXCR7 axis

**Figure 5:**
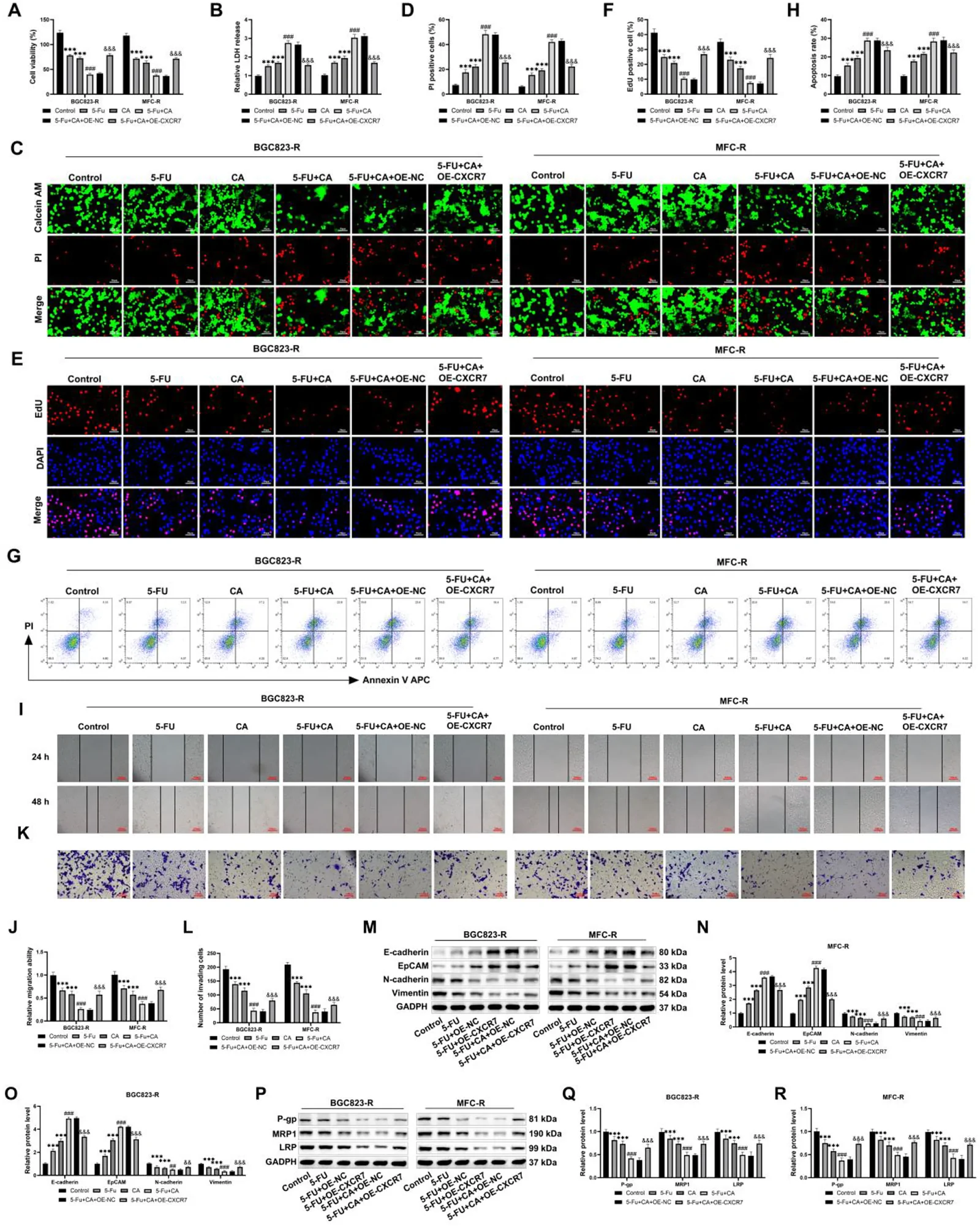
CXCR7 overexpression attenuates the inhibitory effects of CA combined with 5-FU on 5-FU-resistant GC cells. **(A, B)** CA combined with 5-FU reduced cell viability and increased LDH release, whereas CXCR7 overexpression partially restored cell viability and reduced LDH release. **(C–H)** Calcein AM/PI staining, EdU assays, and flow cytometric analysis showed that CXCR7 overexpression reduced 5-FU+CA-induced cell death and apoptosis and partially restored cell proliferation. **(I–L)** Wound healing and Transwell assays revealed that the migratory and invasive capacities of BGC823-R and MFC-R cells suppressed by 5-FU+CA treatment were partially restored by CXCR7 overexpression. **(M–O)** Western blot analysis demonstrated that CXCR7 overexpression partially reversed the effects of 5-FU + CA on EMT-associated proteins. **(P–R)** Western blot analysis showed that CXCR7 overexpression partially restored the expression of the drug resistance-related proteins P-gp, MRP1, and LRP. Data are presented as the mean ± SD. ^***^P < 0.001 *vs.* Control; ^###^*P* < 0.001 *vs.* 5-FU; ^&&&^*P* < 0.001 *vs.* 5-FU+CA+OE-NC.

To verify whether the CA-mediated enhancement of 5-FU sensitivity depends on inhibition of the CXCL12/CXCR7 axis, CXCR7 overexpression rescue experiments were performed in the presence of 5-FU+CA treatment. CCK-8 analysis showed that the 5-FU+CA-mediated suppression of cell viability was partially reversed by forced CXCR7 expression (**Figure 5A**). Meanwhile, 5-FU+CA markedly increased LDH release and the proportion of dead cells. After OE-CXCR7 treatment, LDH release was decreased, with fewer red dead cells and more green live cells observed in Calcein AM/PI staining, suggesting that CXCR7 overexpression attenuated the cytotoxicity induced by CA combined with 5-FU (**Figures 5B–D**). EdU incorporation and flow cytometric assays further revealed that the 5-FU+CA-mediated suppression of proliferation and induction of apoptosis were partially counteracted by CXCR7 overexpression, as evidenced by the recovery of EdU-labeled cells and a decrease in apoptotic frequency (**Figures 5E–H**).

Regarding metastasis-associated phenotypes, the combined treatment with 5-FU and CA markedly reduced wound closure and decreased the number of invading cells; however, these inhibitory effects were partially reversed by ectopic CXCR7 expression, which restored both migratory and invasive capacities (**Figures 5I–L**). In addition, 5-FU+CA promoted an epithelial-like shift, characterized by upregulation of E-cadherin and EpCAM and downregulation of the mesenchymal markers N-cadherin and Vimentin. These changes were partially reversed by CXCR7 overexpression, indicating partial restoration of EMT-associated protein expression (**Figures 5M–O**). Furthermore, CXCR7 overexpression partially relieved the inhibitory effects of 5-FU+CA on drug resistance-related proteins, including P-gp, MRP1, and LRP (**Figures 5P–R**).

Collectively, these results indicate that the enhanced inhibitory effects of CA on 5-FU-resistant GC cell proliferation, migration, invasion, EMT-associated changes, and drug-resistant phenotypes are closely associated with downregulation of the CXCL12/CXCR7 axis. Reactivation of CXCR7 can partially weaken the anti-resistance effects of CA combined with 5-FU.

### 3.6 CA enhances 5-FU-mediated CD8^+^ T cell function via suppression of the CXCL12/CXCR7 axis

**Figure 6:**
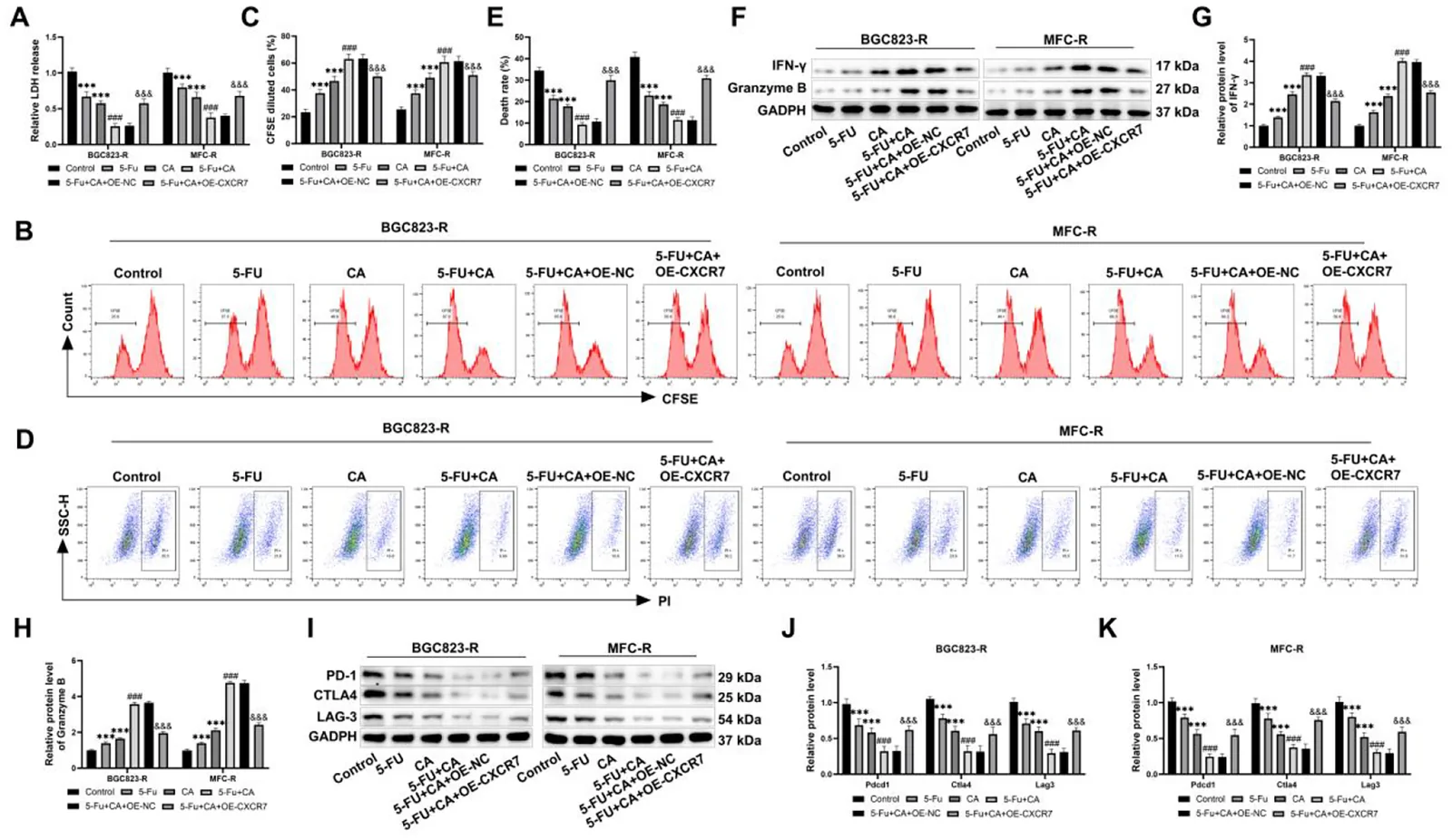
CA combined with 5-FU enhances CD8^+^ T cell function by inhibiting the CXCL12/CXCR7 axis. **(A)** LDH release assays showed that CA combined with 5-FU reduced LDH release from CD8^+^ T cells, whereas CXCR7 overexpression partially reversed this effect. **(B, C)** CFSE assays showed that the combined treatment promoted CD8^+^ T cell proliferation, whereas CXCR7 overexpression reduced the proliferation level. **(D, E)** Flow cytometric analysis showed that CA combined with 5-FU reduced CD8^+^ T cell apoptosis, whereas CXCR7 overexpression partially increased the apoptotic rate of CD8^+^ T cells. **(F–H)** Western blot analysis showed that the combined treatment upregulated IFN-γ and Granzyme B protein expression in CD8^+^ T cells, whereas CXCR7 overexpression reduced the expression of these effector molecules. **(I–K)** Western blot analysis showed that CA combined with 5-FU downregulated PD-1, CTLA4, and LAG-3 expression in CD8^+^ T cells, whereas CXCR7 overexpression partially restored the levels of these inhibitory molecules. Data are presented as the mean ± SD. ^***^P < 0.001 *vs.* Control; ^###^*P* < 0.001 *vs.* 5-FU; ^&&&^*P* < 0.001 *vs.* 5-FU+CA+OE-NC.

To assess whether the CXCL12/CXCR7 axis contributes to the CA/5-FU-induced improvement of CD8^+^ T cell function, co-culture assays were performed using CD8^+^ T cells and 5-FU-resistant GC cells with CXCR7 overexpression. LDH release assays showed that 5-FU+CA significantly reduced LDH release from CD8^+^ T cells, suggesting decreased CD8^+^ T cell injury under co-culture conditions. However, CXCR7 overexpression partially reversed this effect and increased CD8^+^ T cell LDH release, indicating that activation of the CXCL12/CXCR7 axis weakened the protective effect of CA combined with 5-FU on CD8^+^ T cells (**Figure 6A**).

CFSE assays showed that 5-FU+CA promoted CD8^+^ T cell proliferation, whereas the proliferative response was decreased after CXCR7 overexpression (**Figures 6B, C**). Meanwhile, flow cytometric analysis showed that 5-FU+CA reduced the apoptosis rate of CD8^+^ T cells, while OE-CXCR7 partially restored CD8^+^ T cell apoptosis, indicating that CXCR7 activation impaired the survival advantage of CD8^+^ T cells induced by combined treatment (**Figures 6D, E**).

The effector function of CD8^+^ T cells was then evaluated by Western blotting. The results showed that 5-FU+CA upregulated IFN-γ and Granzyme B protein expression in CD8^+^ T cells, whereas CXCR7 overexpression reduced the expression of these effector molecules (**Figures 6F–H**). In addition, 5-FU+CA downregulated the protein levels of the inhibitory receptors PD-1, CTLA4, and LAG-3 in CD8^+^ T cells, while CXCR7 overexpression partially restored their expression levels (**Figures 6I–K**).

Together, these results indicate that CA combined with 5-FU improves CD8^+^ T cell function in the co-culture system by suppressing the CXCL12/CXCR7 axis in 5-FU-resistant GC cells. This effect was characterized by reduced CD8^+^ T cell injury and apoptosis, enhanced proliferative capacity, increased IFN-γ and Granzyme B expression, and decreased inhibitory receptor expression.

### 3.7 CA Enhances the *In Vivo* Antitumor Efficacy of 5-FU and Promotes CD8^+^ T cell antitumor response via suppression of the CXCL12/CXCR7 axis

**Figure 7:**
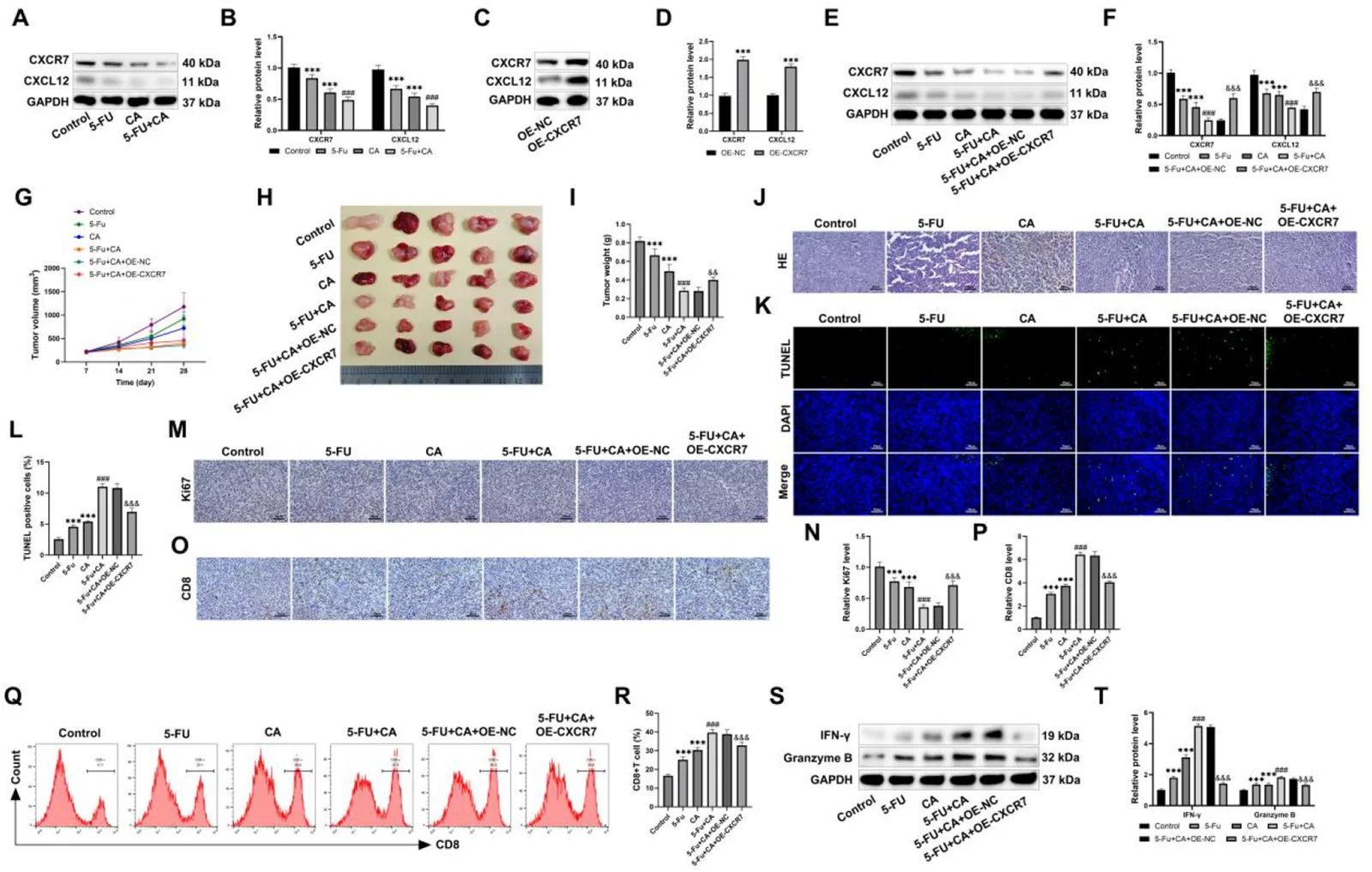
CA enhances the *in vivo* antitumor effect of 5-FU and promotes CD8^+^ T cell antitumor response by suppressing the CXCL12/CXCR7 axis. **(A, B)** Treatment with 5-FU+CA reduced the protein expression of CXCR7 and CXCL12 in tumor tissues. **(C, D)** Increased expression of CXCR7 and CXCL12 in the OE-CXCR7 group indicated successful establishment of the *in vivo* overexpression model. **(E, F)** On the basis of 5-FU+CA treatment, CXCR7 overexpression partially restored CXCR7 and CXCL12 protein expression. **(G–I)** Tumor growth curves, tumor images, and tumor weight results showed that 5-FU+CA significantly suppressed tumor growth, whereas CXCR7 overexpression weakened this tumor-inhibitory effect. **(J–L)** H&E and TUNEL staining indicated that 5-FU+CA aggravated tumor tissue damage and promoted cell apoptosis, whereas apoptosis was reduced after CXCR7 overexpression. **(M, N)** Ki67 immunohistochemistry showed that 5-FU+CA reduced tumor cell proliferation, whereas CXCR7 overexpression increased Ki67 expression. **(O–R)** CD8 immunohistochemistry and flow cytometric analysis showed that 5-FU+CA increased CD8^+^ cell infiltration and the proportion of CD8^+^ cells in tumor tissues, whereas CXCR7 overexpression attenuated this effect. **(S, T)** 5-FU+CA upregulated IFN-γ and Granzyme B expression, whereas CXCR7 overexpression reduced the levels of these effector molecules. Data are presented as the mean ± SD. ^***^P < 0.001 *vs.* Control; ^###^*P* < 0.001 *vs.* 5-FU; ^&&&^*P* < 0.001 *vs.* 5-FU+CA+OE-NC.

To verify whether the *in vitro* findings could be reproduced *in vivo*, an MFC-R cell-derived subcutaneous tumor model was established in 615 mice. Compared with 5-FU monotherapy, the combined administration of CA and 5-FU more markedly reduced CXCR7 and CXCL12 protein expression in tumor tissues, suggesting that CA suppressed the CXCL12/CXCR7 axis *in vivo* (**Figures 7A, B**). In the CXCR7 overexpression model, CXCR7 and CXCL12 expression levels were markedly increased in the OE-CXCR7 group, confirming successful establishment of the overexpression model (**Figures 7C, D**). Further analysis showed that CXCR7 overexpression partially restored CXCR7 and CXCL12 protein expression under 5-FU+CA treatment (**Figures 7E, F**).

Tumor growth curves, endpoint tumor images, and tumor weight measurements showed that either 5-FU or CA alone inhibited tumor growth to some extent, whereas combined treatment with 5-FU and CA exerted a more pronounced antitumor effect. After CXCR7 overexpression, tumor volume and tumor weight were increased compared with the 5-FU+CA+OE-NC group, indicating that CXCR7 overexpression weakened the *in vivo* antitumor efficacy of CA combined with 5-FU (**Figures 7G–I**). Histological analysis showed that 5-FU+CA induced more evident tumor tissue damage and increased the proportion of TUNEL-positive cells, while reducing Ki67 expression. These changes were partially reversed by CXCR7 overexpression (**Figures 7J–N**).

Further analysis of tumor immune infiltration showed that 5-FU+CA markedly increased CD8^+^ cell infiltration and the proportion of CD8^+^ cells in tumor tissues, whereas these effects were attenuated by CXCR7 overexpression (**Figures 7O–R**). Meanwhile, 5-FU+CA upregulated IFN-γ and Granzyme B expression in tumor tissues, suggesting enhanced CD8^+^ T cell-related effector activity; however, the expression of these molecules was reduced after CXCR7 overexpression (**Figures 7S, T**).

In summary, these results indicate that CA enhances the *in vivo* antitumor response to 5-FU by suppressing the CXCL12/CXCR7 axis, accompanied by increased CD8^+^ T cell infiltration and enhanced antitumor activity.

### 3.8 CA plus 5-FU potentiates the *in vivo* antitumor effect of anti-PD-L1 therapy

**Figure 8:**
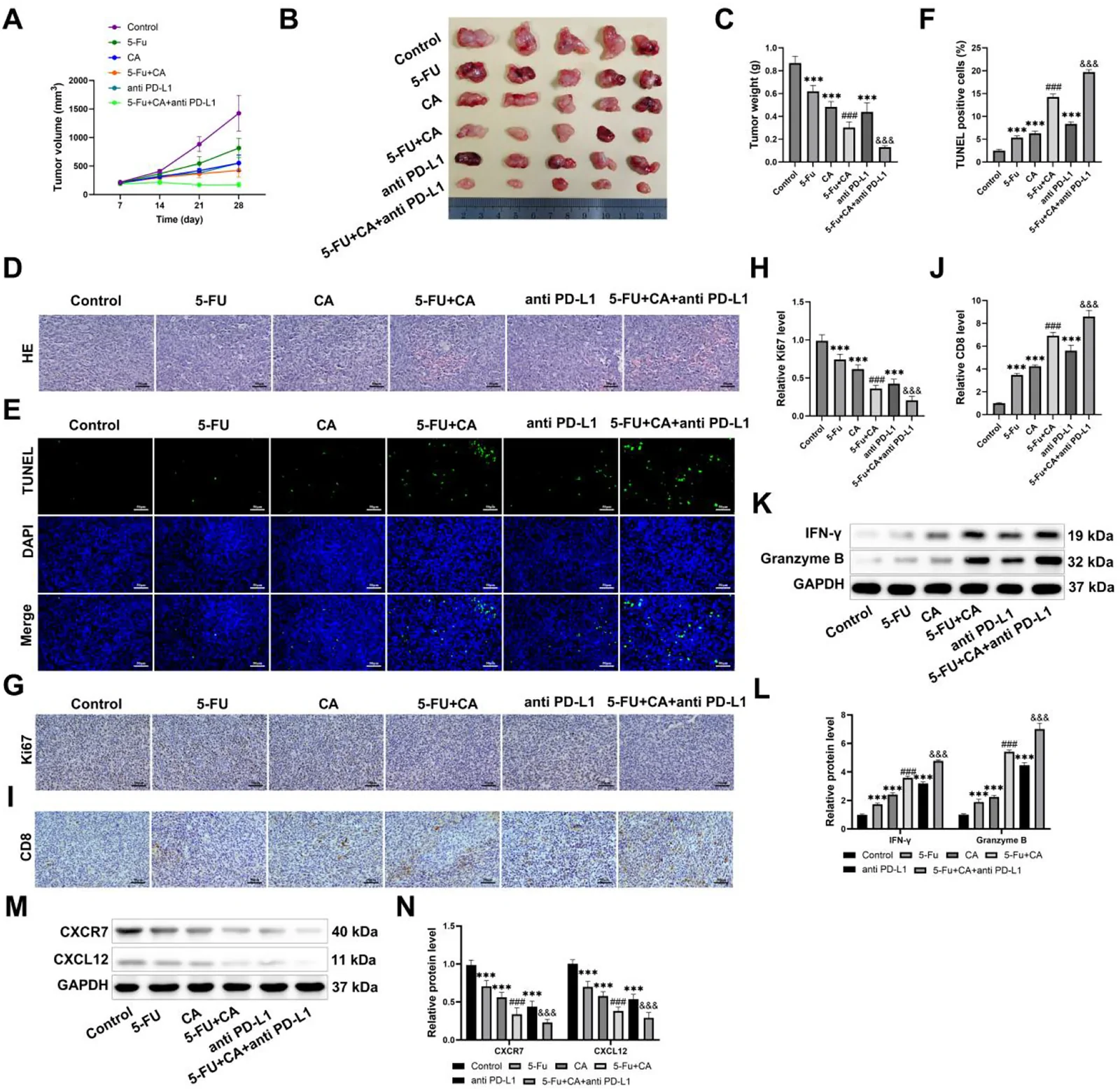
CA combined with 5-FU enhances the *in vivo* antitumor efficacy of anti-PD-L1 treatment. **(A–C)** Tumor growth curves, tumor images, and tumor weight analysis showed that 5-FU+CA+anti-PD-L1 further suppressed tumor growth. **(D)** H&E staining showed that triple-combination therapy induced more evident tumor tissue damage. **(E, F)** TUNEL staining showed that 5-FU+CA+anti-PD-L1 promoted tumor cell apoptosis. **(G, H)** Ki67 immunohistochemistry showed that triple-combination therapy further reduced tumor cell proliferation. **(I, J)** CD8 immunohistochemistry revealed that 5-FU+CA+anti-PD-L1 increased CD8^+^ cell infiltration in tumor tissues. **(K, L)** Western blot analysis showed that triple-combination therapy upregulated IFN-γ and Granzyme B expression, suggesting an enhanced antitumor effector response. **(M, N)** Western blot analysis showed that triple-combination therapy downregulated CXCL12 and CXCR7 expression. Data are presented as the mean ± SD. ^***^P < 0.001 *vs.* Control; ^###^*P* < 0.001 *vs.* 5-FU; ^&&&^*P* < 0.001 *vs.* anti-PD-L1.

Given that CA combined with 5-FU improved CD8^+^ T cell function and reduced the immunosuppressive phenotype of resistant GC cells, we further evaluated its effect on the *in vivo* therapeutic efficacy of anti-PD-L1. A subcutaneous tumor model was established using MFC-R cells. Tumor volume, endpoint tumor images, and tumor weight measurements showed that both anti-PD-L1 monotherapy and 5-FU+CA inhibited tumor growth, whereas the 5-FU+CA+anti-PD-L1 group exhibited the most pronounced tumor-suppressive effect, with lower tumor volume and weight than the anti-PD-L1 group (**Figures 8A–C**).

Histological analysis showed that tumor tissue damage was more evident in the triple-combination treatment group. The proportion of TUNEL-positive cells was increased, whereas Ki67 expression was further decreased, suggesting that 5-FU+CA enhanced anti-PD-L1-induced tumor damage, apoptosis, and proliferation inhibition (**Figures 8D–H**). Subsequent immunological assessments revealed that the triple combination of 5-FU, CA, and anti-PD-L1 significantly increased CD8^+^ cell infiltration and upregulated IFN-γ and Granzyme B expression compared with anti-PD-L1 monotherapy, suggesting an enhanced CD8^+^ cell-related effector response (**Figures 8I–L**). In addition, Western blot analysis showed that triple-combination therapy markedly reduced CXCL12 and CXCR7 expression in tumor tissues compared with anti-PD-L1 monotherapy, indicating suppression of the CXCL12/CXCR7 axis associated with an immunosuppressive tumor microenvironment and therapeutic resistance (**Figures 8M, N**).

Collectively, these results indicate that CA combined with 5-FU enhances the *in vivo* antitumor efficacy of anti-PD-L1 therapy, which may be associated with increased CD8^+^ cell infiltration and enhanced antitumor immune activity.

### 3.9 CA enhances 5-FU-mediated suppression of EMT and tumor metastasis *in vivo* via CXCL12/CXCR7 axis inhibition

**Figure 9:**
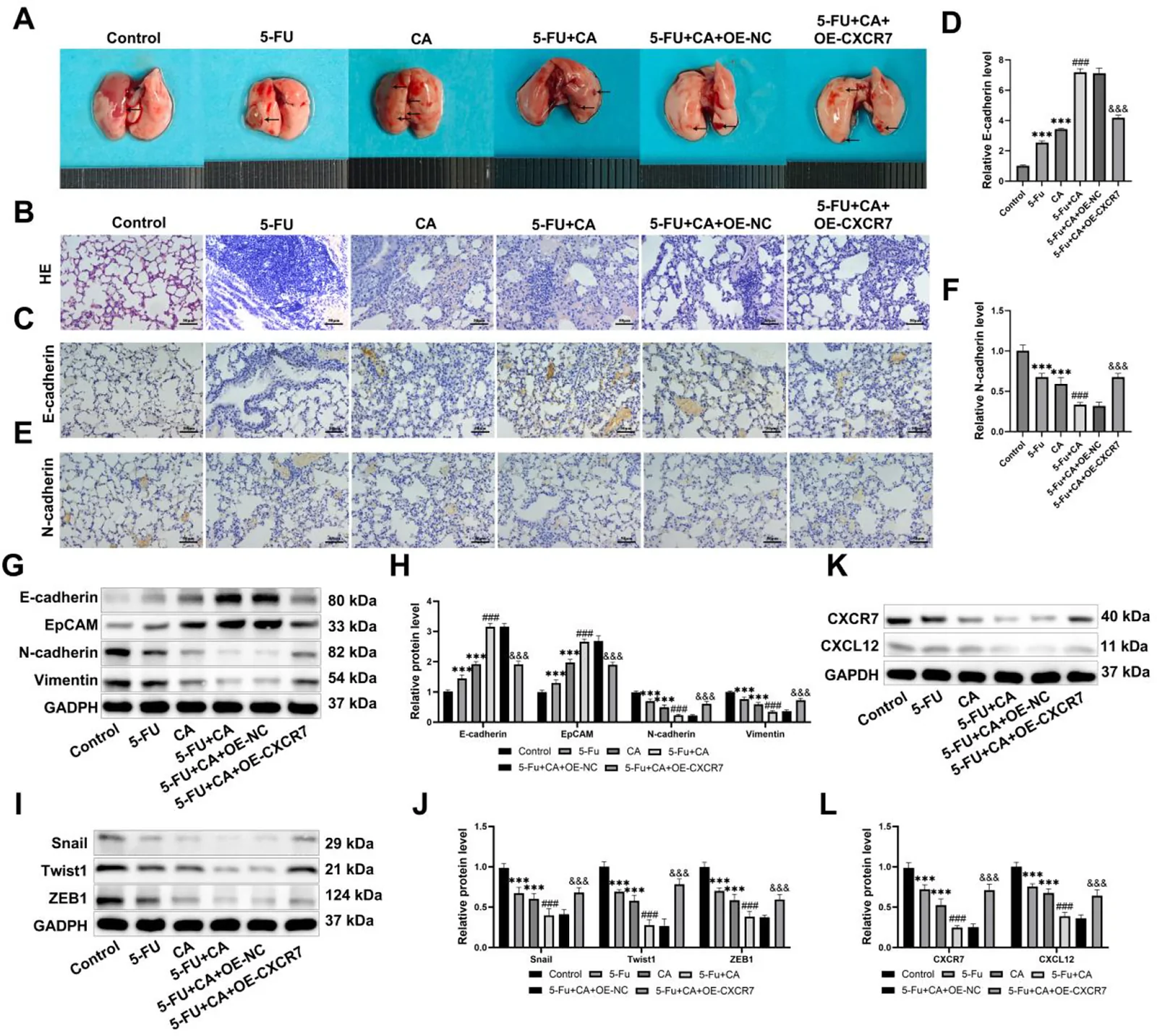
CA enhances the inhibitory effects of 5-FU on GC lung metastasis and EMT by suppressing the CXCL12/CXCR7 axis. **(A, B)** Gross images of lung tissues and H&E staining showed that 5-FU+CA reduced the formation of pulmonary metastatic nodules and metastatic lesions, whereas CXCR7 overexpression partially weakened this anti-metastatic effect. **(C–F)** Immunohistochemical analysis revealed that 5-FU+CA increased E-cadherin expression and reduced N-cadherin expression, whereas CXCR7 overexpression partially reversed these EMT-associated marker changes. **(G, H)** Western blot analysis confirmed that 5-FU+CA elevated E-cadherin and EpCAM levels while reducing N-cadherin and Vimentin levels; these changes were partially counteracted by CXCR7 overexpression. **(I, J)** 5-FU+CA downregulated Snail, Twist1, and ZEB1 expression, whereas CXCR7 overexpression partially restored the levels of these EMT-related transcription factors. **(K, L)** 5-FU+CA inhibited CXCR7 and CXCL12 expression in lung metastatic tissues, whereas CXCR7 overexpression partially restored CXCR7 and CXCL12 protein expression. Data are presented as the mean ± SD. ^***^P < 0.001 *vs.* Control; ^###^*P* < 0.001 *vs.* 5-FU; ^&&&^*P* < 0.001 *vs.* 5-FU+CA+OE-NC.

To assess the influence of CA on 5-FU’s anti-metastatic efficacy, we established a pulmonary metastasis model via tail vein inoculation of MFC-R cells. Both macroscopic inspection and histological H&E analysis revealed that 5-FU monotherapy diminished the frequency of surface nodules and internal pulmonary lesions. Notably, the overall metastatic burden was even more substantially attenuated in the 5-FU+CA group. In contrast, lung metastatic nodules and lesions increased again after CXCR7 overexpression, suggesting that CXCR7 activation weakened the *in vivo* anti-metastatic effect of CA combined with 5-FU (**Figures 9A, B**).

Immunohistochemical analysis showed that 5-FU+CA increased E-cadherin expression and decreased N-cadherin expression, whereas CXCR7 overexpression reversed these changes, indicating partial reversal of EMT-associated marker changes (**Figures 9C–F**). Western blot analysis further confirmed that 5-FU+CA upregulated E-cadherin and EpCAM, downregulated N-cadherin and Vimentin, and reduced the expression of Snail, Twist1, and ZEB1. In contrast, OE-CXCR7 partially restored the levels of mesenchymal markers and EMT transcription factors (**Figures 9G–J**). Meanwhile, 5-FU+CA inhibited CXCL12/CXCR7 axis expression in lung metastatic tissues, whereas CXCR7 overexpression partially restored CXCR7 and CXCL12 protein expression (**Figures 9K, L**).

In aggregate, these findings suggest that by inhibiting both the CXCL12/CXCR7 signaling axis and the progression of EMT, CA strengthens the ability of 5-FU to suppress the *in vivo* metastasis of 5-FU-resistant GC cells.

### 3.10 CA exhibits a favorable safety profile in mice

**Figure 10:**
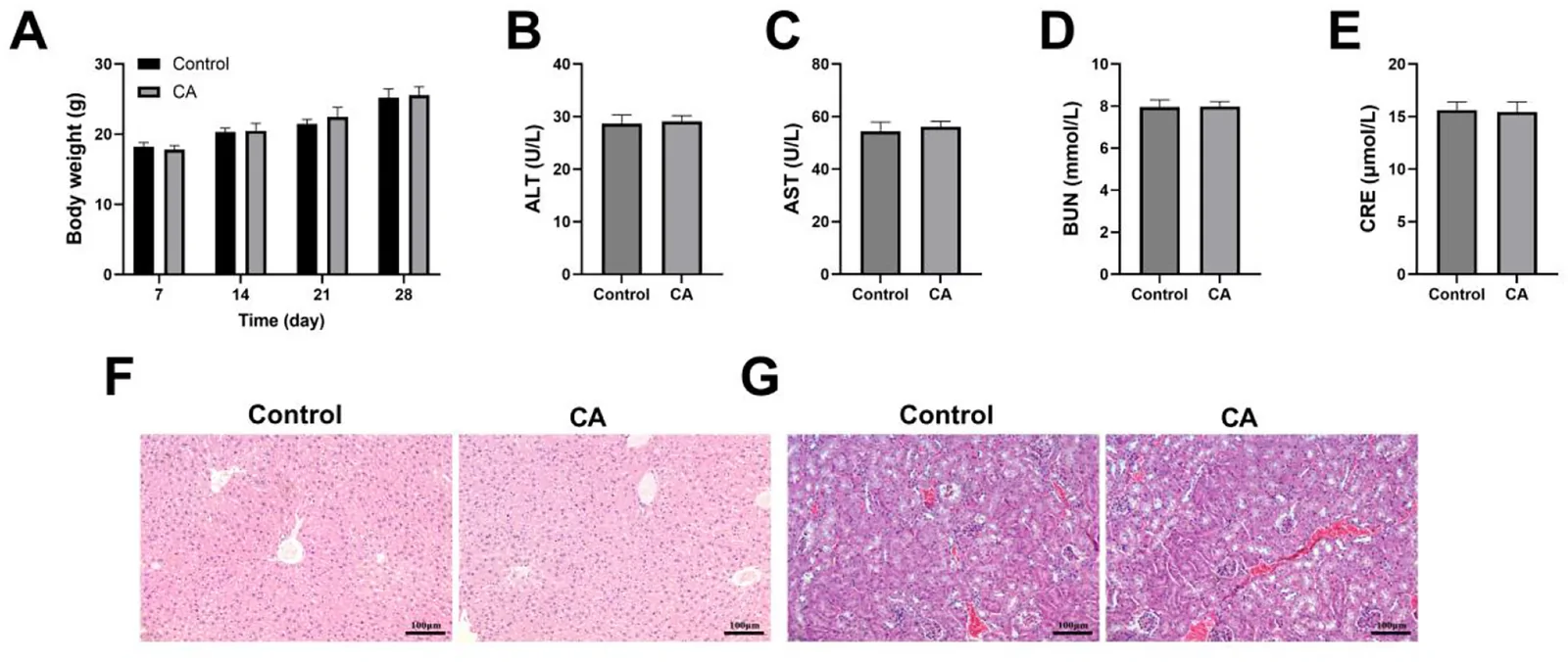
CA shows no obvious toxicity in mice at the tested dose. **(A)** Body weight monitoring over 4 weeks showed that CA treatment did not cause obvious body weight loss. **(B–E)** Serum ALT, AST, BUN, and CRE levels showed no marked differences between the CA and Control groups, suggesting that liver and kidney function were not significantly affected. **(F, G)** H&E staining of liver and kidney tissues showed no obvious structural disruption or pathological injury.

To evaluate the *in vivo* safety of CA, Control and CA groups were established. Mouse body weight was monitored, and liver and kidney function indicators and pathological changes were assessed. The results showed that, during the 4-week observation period, the trend in body weight change in the CA group was generally consistent with that in the Control group, with no obvious weight loss or abnormal emaciation observed (**Figure 10A**). Serum biochemical analysis showed that the levels of ALT, AST, BUN, and CRE in the CA group were not markedly increased compared with those in the Control group, suggesting that CA did not cause obvious liver or kidney dysfunction (**Figures 10B–E**). H&E staining showed that mice in the CA group exhibited intact liver tissue architecture, regularly arranged hepatocytes, and clear glomerular and tubular structures, with no obvious inflammatory infiltration, necrosis, steatosis, or renal pathological damage (**Figures 10F, G**).

Altogether, CA did not induce obvious body weight loss, liver or kidney dysfunction, or pathological damage in liver and kidney tissues, suggesting a favorable *in vivo* safety profile at the tested dose.

## 4 Discussion

Chemoresistance in GC is characterized not only by reduced drug sensitivity but also by sustained proliferation, evasion of apoptosis, enhanced migration and invasion, and activation of EMT (8, 35). Previous studies have demonstrated that 5-FU-resistant GC cells can maintain a resistant phenotype by increasing proliferative capacity, suppressing apoptosis, and upregulating drug resistance-related proteins (36). In accordance with these observations, our data demonstrated that CA bolstered the suppressive activity of 5-FU against 5-FU-resistant GC cells. This was manifested as diminished cell viability and proliferation, alongside elevated cellular damage and apoptosis. Such findings imply that CA sensitizes resistant cells to 5-FU and intensifies its cytotoxic impact.

EMT is an important mechanism by which tumor cells acquire invasive and metastatic capacities as well as therapeutic tolerance (37). It has been reported that EMT is closely associated with migration, invasion, and chemoresistance in GC cells, and tumor cells undergoing EMT often exhibit stronger metastatic potential and resistance to apoptosis (38). Building on these findings, the current investigation subsequently assessed the impact of CA/5-FU co-administration on the aggressive properties of refractory cells, particularly regarding migration, invasion, EMT-related markers, and anoikis. The results demonstrated that the synergistic application of CA and 5-FU suppressed the migratory, invasive, and EMT-related activities of 5-FU-resistant GC cells, whereas concurrently fostering anoikis. This suggests that CA both strengthens 5-FU’s cytotoxic impact and mitigates the metastatic-related malignant features of these resistant cells.

CXCL12 is an important ligand of CXCR7. Through binding to CXCL12, CXCR7 can influence its local concentration, spatial distribution, and related downstream signaling, thereby participating in the regulation of CXCL12-mediated cellular behaviors (39). The CXCL12/CXCR7 signaling axis is documented to facilitate tumor progression across multiple cancers by accelerating the proliferation and motility of malignant cells, driving angiogenesis, establishing an immune-evasive microenvironment, and fostering the development of drug resistance (20). Mechanistically, accumulating evidence indicates that STAT3 and PI3K/Akt are important downstream signaling pathways associated with CXCL12/CXCR7 activation. Guo et al. demonstrated that CXCL12/CXCR7 activation promoted EMT and metastatic behavior in esophageal cancer through STAT3 signaling, whereas inhibition of STAT3 attenuated these effects (40). CXCR7 has also been linked to PI3K/Akt signaling, as CXCR7 knockdown suppressed PI3K/Akt and β-arrestin signaling together with tumor cell proliferation and invasion (41). These tumor-intrinsic signaling events may also intersect with immune regulation. In GC, Chen et al. showed that CXCL12 contributed to anti-PD-1 resistance, while CXCL12 knockdown increased intratumoral CD8^+^ T cells and IFN-γ^+^ cells and reduced MDSC accumulation (42). Consistently, PI3K/Akt/HIF1A signaling has been implicated in CD8^+^ T cell exhaustion and PD-L1 upregulation, indicating that this pathway may provide a mechanistic link between oncogenic signaling and impaired antitumor T cell function (43). Together, these findings suggest that CXCL12/CXCR7-associated signaling may link EMT- and resistance-related tumor phenotypes with an immunosuppressive microenvironment, thereby integrating chemoresistance and impaired CD8^+^ T cell function within a coordinated tumor-adaptive response. Consistent with this mechanistic framework, the current research identified significantly elevated expression of CXCR7 and CXCL12 in 5-FU-resistant GC cells, implying that this signaling axis contributes to the preservation of the chemoresistant phenotype. Subsequent experiments revealed that CXCR7 up-regulation facilitated survival, proliferation, and motility in resistant cells subjected to 5-FU, while simultaneously increasing the expression of resistance-related markers such as P-gp, MRP1, and LRP. Conversely, CA combined with 5-FU inhibited CXCR7 and CXCL12 expression, and Co-IP confirmed the interaction between these two proteins. CXCR7 overexpression partially weakened the regulatory effects of CA plus 5-FU on the resistant phenotype, EMT, and apoptosis. These findings further support the functional involvement of the CXCL12/CXCR7 axis in CA-mediated 5-FU sensitization, potentially involving STAT3- and PI3K/Akt-related signaling.

Although CD8^+^ T cells serve as the primary executors of antitumor immunity, their activity is frequently compromised within chemoresistant tumor microenvironments (44). Existing literature suggests that augmenting the recruitment of CD8^+^ T cells and reviving their functionality can boost immune-mediated tumor cell clearance (45). Accordingly, the current investigation conducted a deeper evaluation of how CA plus 5-FU influences CD8^+^ T cell functionality and markers related to immunosuppression. Our findings demonstrated that CA in conjunction with 5-FU stimulated CD8^+^ T cell expansion and the production of effector molecules, attenuated CD8^+^ T cell apoptosis, and simultaneously lowered the levels of tumor cell PD-L1 as well as inhibitory markers such as PD-1, CTLA4, and LAG-3. CXCR7 overexpression partially weakened these immune-enhancing effects, suggesting that the CXCL12/CXCR7 axis may contribute to immune escape by affecting the interaction between tumor cells and CD8^+^ T cells, in addition to promoting 5-FU resistance. Therefore, CA combined with 5-FU may enhance immune-mediated killing of drug-resistant GC cells by restoring CD8^+^ T cell function and reducing the expression of immunosuppressive molecules.

This study further confirmed the comprehensive regulatory impacts of CA and 5-FU co-treatment on tumor expansion, metastatic spread, and immune-mediated defense within an *in vivo* setting. Our data demonstrated that CA alongside 5-FU inhibited the development of MFC-R subcutaneous tumor and pulmonary metastasis, stimulated apoptotic cell death, bolstered CD8^+^ T cell recruitment along with IFN-γ and Granzyme B levels, and refined the *in vivo* anti-tumor effectiveness of anti-PD-L1 therapy. In contrast, CXCR7 overexpression partially weakened these antitumor and anti-metastatic effects, supporting the CXCL12/CXCR7 axis as an important target through which CA exerts its effects. Safety evaluation showed that CA did not cause obvious body weight loss, liver or kidney dysfunction, or pathological damage to major organs, suggesting favorable *in vivo* tolerability. Taken together, these *in vivo* findings further strengthen the experimental evidence supporting the antitumor effects of CA combined with 5-FU from the perspectives of tumor burden, metastatic capacity, immune response, and safety.

Although these findings provide a mechanistic basis for the chemosensitizing and immunomodulatory effects of CA in 5-FU-resistant GC, some aspects still require further investigation. The present study mainly focused on the CXCL12/CXCR7 axis, but the upstream events through which CA regulates this pathway remain incompletely defined. Although previous studies implicate STAT3 and PI3K/Akt as downstream pathways of CXCL12/CXCR7 signaling, their activation and functional contribution were not directly examined in the present study. In addition, the experimental evidence was primarily obtained from established resistant cell models and mouse tumor models, and further validation using patient-derived samples, organoids, or clinically relevant drug-resistant GC tissues would help strengthen the translational significance of these findings. Moreover, while the current data suggest that CA enhances CD8^+^ T cell-related antitumor responses, a more comprehensive characterization of immune cell populations within the tumor microenvironment may provide deeper insight into its immunoregulatory effects. Future studies should also evaluate the pharmacokinetic properties, long-term safety, and optimal dosing strategy of CA when combined with chemotherapy or immunotherapy.

## 5 Conclusion

Our research reveals that CA sensitizes chemoresistant GC cells to 5-FU through the suppression of pathologically active CXCL12/CXCR7 signaling. Additionally, it curtails EMT and metastatic traits while bolstering immune-mediated tumor clearance driven by CD8^+^ T cells. CA exhibits a dual regulatory effect involving chemosensitization and immune activation, providing experimental evidence for its potential use as an adjuvant strategy to overcome 5-FU resistance and optimize chemo-immunotherapy in GC.

## Data Availability

The original contributions presented in the study are included in the article/supplementary material, further inquiries can be directed to the corresponding author/s.

## Glossary

5-FU: 5-Fluorouracil
ABCB1: ATP-binding cassette subfamily B member 1
ABCC1: ATP-binding cassette subfamily C member 1
ALT: Alanine aminotransferase
ANOVA: Analysis of variance
AST: Aspartate aminotransferase
BCA: Bicinchoninic acid
BSA: Bovine serum albumin
BUN: Blood urea nitrogen
CA: Carnosic acid
CCK-8: Cell Counting Kit-8
CFDA-SE: Carboxyfluorescein diacetate succinimidyl ester
CFSE: Carboxyfluorescein succinimidyl ester
Co-IP: Co-immunoprecipitation
CRE: Creatinine
CTLA4: Cytotoxic T-lymphocyte-associated protein 4
CXCL12: C-X-C motif chemokine ligand 12
CXCR7: C-X-C chemokine receptor 7
DAB: 3,3′-Diaminobenzidine
DAPI: 4′,6-Diamidino-2-phenylindole
DMSO: Dimethyl sulfoxide
ECL: Enhanced chemiluminescence
EdU: 5-Ethynyl-2′-deoxyuridine
EMT: Epithelial–mesenchymal transition
EpCAM: Epithelial cell adhesion molecule
FBS: Fetal bovine serum
GC: gastric cancer
H&E: Hematoxylin and eosin
HRP: Horseradish peroxidase
IC50: Half-maximal inhibitory concentration
IF: Immunofluorescence
IFN-γ: Interferon-γ
IHC: Immunohistochemistry
LAG-3: Lymphocyte activation gene 3
LDH: Lactate dehydrogenase
LRP: Lung resistance-related protein
MRP1: Multidrug resistance-associated protein 1
OD: Optical density
PBS: Phosphate-buffered saline
PD-1: Programmed death 1
PD-L1: Programmed death ligand 1
PD-1: Programmed cell death protein 1
P-gp: P-glycoprotein
PI: Propidium iodide
PVDF: Polyvinylidene fluoride
RIPA: Radioimmunoprecipitation assay buffer
SD: Standard deviation
SDS-PAGE: Sodium dodecyl sulfate-polyacrylamide gel electrophoresis
SPF: Specific pathogen-free
STR: Short tandem repeat
TUNEL: Terminal deoxynucleotidyl transferase dUTP nick-end labeling.
